# Ultrahigh discharge voltage plateau of 3.2 V achieved in Li-CO_2_ batteries using iron polyphthalocyanine

**DOI:** 10.1126/sciadv.aei5080

**Published:** 2026-10-02

**Authors:** Ning Zhao, Yi Zhang, Li-Min Liu, Han-Qi Zhang, Zheng Meng, Shu-Jiang Ding, Qiang Wang, Xiao-Fei Hu

**Affiliations:** ^1^ School of Chemistry, School of Electrical Engineering, Xi’an Jiaotong University, Xi’an 710061, ShaanXi, P. R. China.; ^2^ State Key Laboratory of Precision and Intelligent Chemistry, School of Chemistry and Materials Science, University of Science and Technology of China, Hefei 230026, Anhui, P. R. China.; ^3^ Engineering Research Center of Energy Storage Materials and Devices, Ministry of Education, National Innovation Platform (Center) for Industry-Education Integration of Energy Storage Technology, Xi’an Jiaotong University, Xi’an 710061, ShaanXi, P. R. China.; ^4^ China Automotive Technology and Research Center Intelligent Technology (Tianjin) Co., Ltd., Tianjin 300300, P. R. China.

## Abstract

Rechargeable lithium-carbon dioxide (Li-CO_2_) batteries offer great potential for greenhouse gas use and high-energy-density storage. However, their practical application is hindered by the formation of a thermodynamically stable Li_2_CO_3_ (lithium carbonate) product that is hard to decompose during charging. Highly crystalline and conductive reticular material iron polyphthalocyanine (pFe^II^Pc) as a solid-state redox mediator into Li-CO_2_ batteries tailors the cathode reaction pathway, enabling stable Li_2_C_2_O_4_ (lithium oxalate) generation and an extraordinary discharge plateau of 3.2 V beyond the theoretical limit. Beyond delivering an ultrahigh discharge capacity of 28,790 milliampere-hours per gram and reversible cyclability over 1400 hours (cutoff capacity of 1000 milliampere-hours per gram) at 100 milliamperes per gram, the Li-CO_2_ batteries exhibit excellent temperature adaptability (−40 to 80°C). The Li-CO_2_ pouch cells using pFe^II^Pc as the solid-state redox mediator achieve a high energy density of 678 watt-hours per kilogram on the basis of total device mass and maintain reversible cycling for 251 hours. Our work pioneers a promising pathway toward practical high-efficiency Li-CO_2_ batteries.

## INTRODUCTION

The excessive consumption on fossil fuels has resulted in an energy crisis and substantial carbon dioxide (CO_2_) emissions, exerting profound adverse impacts on environmental integrity and sustainable economic development (*1*–*5*). Consequently, developing an innovative electrochemical energy storage device with dual energy-saving and emission-reduction capabilities has become an urgent imperative. Recently, the reversible lithium-carbon dioxide (Li-CO_2_) batteries have emerged as promising candidates for next-generation energy storage systems owing to the superb theoretical energy density of 1876 W·hour kg^−1^ and CO_2_ utilization efficiency (*6*–*14*). Furthermore, Li-CO_2_ batteries exhibit unparalleled application potential in CO_2_-rich environments, particularly for deep-sea missions and Mars exploration where atmospheric CO_2_ concentrations exceed 95% (*15*–*18*).

However, Li_2_CO_3_ pathway Li-CO_2_ batteries typically exhibit limited discharge voltages of 2.0 to 2.5 V because of strong CO_2_ bond interactions and sluggish reaction kinetics, notably lower than the theoretical value of 2.8 V (4Li^+^ + 3CO_2_ + 4e^−^ → 2Li_2_CO_3_ + C, *E*^0^ = 2.80 V versus Li^+^/Li) (*19*–*27*). Although the introduction of cobalt phthalocyanine has increased the discharge voltage of the Li-CO_2_ battery via the Li_2_CO_3_-product pathway to 2.98 V, this additive suffers risks of contaminating lithium metal anodes, and the high thermodynamic stability of Li_2_CO_3_ [∆_f_*G*^0^(Li_2_CO_3_) = −1132.1 kJ mol^−1^] results in a large overpotential, necessitating a charging voltage as high as 4.38 V for decomposition (*23*, *28*–*31*). Notably, the theoretical decomposition voltage of side reaction in the Li-CO_2_ battery (Li_2_CO_3_ − 2e^−^ → 2Li^+^ + CO_2_ + ^1^/_2_^1^O_2_, *E*^0^ = 4.1 V versus Li/Li^+^) is 4.1 V, indicating a high probability of parasitic reactions occurring (*32*). The radical oxygen (^1^O_2_) produced by parasitic reactions may can aggressively degrade both electrodes and the electrolyte, ultimately leading to abrupt death of Li-CO_2_ batteries.

In contrast to the Li_2_CO_3_ pathway (2.8 V), the Li_2_C_2_O_4_-product route (3.01 V) exhibits superior theoretical discharge voltage and faster reaction kinetics in the Li-CO_2_ battery (*33*, *34*). Also, Li_2_C_2_O_4_ exhibits both lower thermodynamic stability (∆_f_*G*^0^ = −286 kJ mol^−1^) and diminished decomposition energy barriers (*E*_a_ = 0.75 eV) relative to Li_2_CO_3_ (*E*_a_ = 2.1 eV). As a result, Li-CO_2_ batteries following the Li_2_C_2_O_4_-product route deliver high energy efficiency and extended cycle life (*30*, *35*, *36*). Attractively, recent studies have confirmed that heterogeneous catalysts (e.g., Mo- and Ti-based materials) and homogeneous catalysts (e.g., Cu- and Ru-based complexes) can ensure the stable formation of Li_2_C_2_O_4_ in the Li-CO_2_ battery through a two-electron reaction (2Li^+^ + 2CO_2_ + 2e^−^ ↔ Li_2_C_2_O_4_, *E*^0^ = 3.01 V versus Li/Li^+^) (*33*–*35*, *37*, *38*). On the one hand, the use of heterogeneous catalysts enables stable formation of Li_2_C_2_O_4_ in Li-CO_2_ batteries, but the practical discharge voltage (2.8 V) of the Li_2_C_2_O_4_-product route Li-CO_2_ battery remains below the theoretical value (3.01 V), and further improvements in cycle life are still required (*39*). On the other hand, homogeneous catalysts (soluble redox mediators) demonstrably elevate the discharge voltage plateau to ∼3.0 V, yet the underlying mechanism for this high-voltage platform remains unclear. Homogeneous catalysts inherently suffer from inevitable critical limitations, including poor solubility, sluggish kinetics, severe corrosion of lithium anodes, and pronounced self-decomposition, which severely restrict the practical application of homogeneous catalysts in Li-CO_2_ batteries (*38*, *40*, *41*). Accordingly, the rational design of high-efficiency solid-state redox mediators represents a promising strategy for elevating the discharge voltage plateau (to >3.01 V) in the Li_2_C_2_O_4_-product route Li-CO_2_ battery.

Herein, a highly crystalline and conductive reticular material iron polyphthalocyanine (pFe^II^Pc) was introduced as a solid-state redox mediator for Li-CO_2_ batteries (*42*). As shown in Fig. 1A, using pFe^II^Pc as the solid-state redox mediator in Li-CO_2_ batteries not only achieves stable generation of Li_2_C_2_O_4_ but also boosts the discharge voltage to 3.2 V, exceeding theoretical limit. The solid-state redox mediator pFe^II^Pc features a conductive and stable network structure, along with precisely distributed single-active sites and multiactive centers. Owing to pFe^II^Pc’s ultrahigh electron affinity and excellent stabilizing effect on Li_2_C_2_O_4_, pFe^II^Pc preferentially accepts electrons instead of CO_2_ and then transfers the captured electrons to CO_2_ to form CO_2_^−^ (activation) while simultaneously adsorbing Li^+^ ions (Fig. 1, B and C). Ultimately, the monomers undergo coupled catalysis to form the final product Li_2_C_2_O_4_, which is stabilized by pFe^II^Pc. Therefore, the Li-CO_2_ battery with pFe^II^Pc as the solid-state redox mediator exhibits an ultrahigh discharge voltage plateau of 3.2 V, an exceptional discharge capacity of 28,790 mA·hour g^−1^ with a reversible charge capacity of 26,338 mA·hour·g^−1^, a low voltage gap of 0.58 V, a long cycling life exceeding 1480 hours, and ultrawide temperature range operation (−40° to 80°C). As a proof of concept, the Li-CO_2_ pouch cells achieve an energy density of 678 W·hour kg^−1^ on the basis of the total device mass and demonstrate stable cycling performance. This work is expected to accelerate the practical application of Li-CO_2_ batteries.

**Fig. 1.**
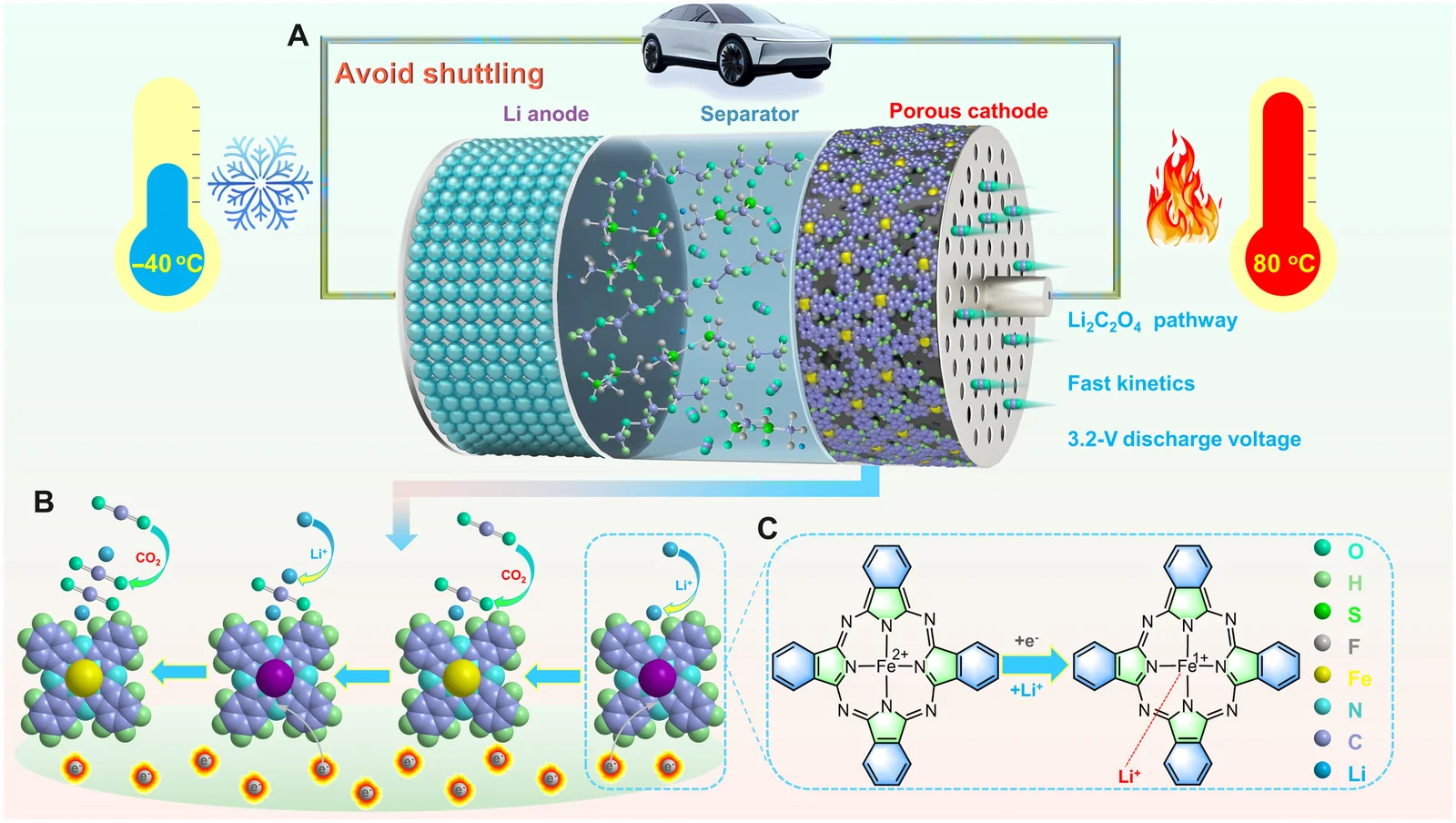
Schematic illustration of the reaction mechanism. (**A**) Model of the Li-CO_2_ battery with pFe^II^Pc as the solid-state redox mediator. (**B**) Solid-state pFe^II^Pc-mediated reaction mechanism toward the Li_2_C_2_O_4_ route. (**C**) Process of pFe^II^Pc gaining electrons and adsorbing Li^+^.

## RESULTS

### Electrochemical performance of the Li-CO_2_ battery catalyzed by pFe^II^Pc

Highly crystalline and conductive pFe^II^Pc has been successfully synthesized through the annulation of tetracyanobenzene in the presence of transition metals (fig. S1). A typical three-electrode cell with a controlled atmosphere was used to investigate the catalytic activity of the pFe^II^Pc cathode toward the CO_2_ reduction reaction. As shown in Fig. 2A and fig. S2, under an Ar atmosphere, the solid-state redox mediator pFe^II^Pc exhibits a pair of redox peaks at 3.23 V (*E*_d1_) and 3.45 V (*E*_c1_), which suggests the possible occurrence of a reversible redox reaction between Fe(II) and Fe(I) in the solid-state redox mediator pFe^II^Pc. Similarly, a reduction peak observed at the same position (3.23 V, *E*_d2_) on the cyclic voltammetry (CV) curve indicates that the same reduction process occurs in the solid-state redox mediator pFe^II^Pc under a CO_2_ atmosphere. This can be ascribed to the reduction reaction of pFe^II^Pc and pFe^I^Pc, as traced in Eq. 1. In addition, compared to the oxidation peak observed in Ar, the oxidation peak in CO_2_ appears at 3.75 V (*E*_c2_), which is attributed to the decomposition of the discharge product Li_2_C_2_O_4_, facilitated by the electron-transfer cascade from pFe^I^Pc to CO_2_ during Li_2_C_2_O_4_ formation. Both in situ online gas chromatography–mass spectrometry and experimental results demonstrate that pFe^II^Pc, after gaining electrons and being reduced to pFe^I^Pc, can promote the CO_2_ reduction reaction (figs. S3 to S5). As shown in fig. S6, ultraviolet-visible spectroscopy shows no notable shift in the spectral features after discharge in CO_2_, while a blue shift in the characteristic peaks is observed after discharge in Ar, which aligns with the expected reduction of pFe^II^Pc to pFe^I^Pc (*43*). By comparing the above results, it can be concluded that in the presence of the solid-state redox mediator pFe^II^Pc, the reaction pathway of the Li-CO_2_ battery is altered by the transfer of electrons from pFe^I^Pc to CO_2_.

$${\text{pFe}}^{\text{II}}\text{Pc}+e^{-}↔{\text{pFe}}^{I}\text{Pc}$$ (1)

**Fig. 2.**
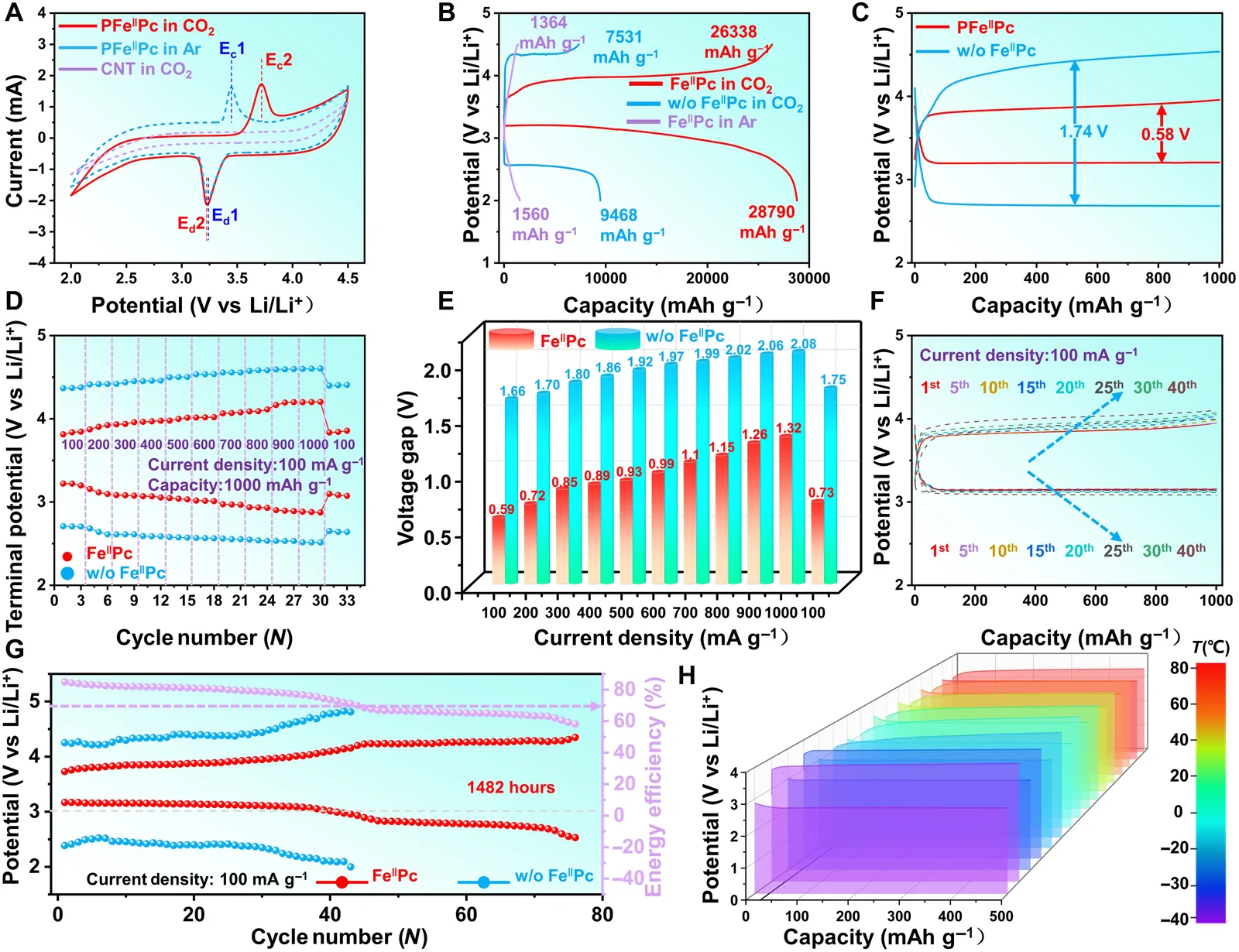
Electrochemical performance characterizations of the pFe^II^Pc cathode for Li-CO_2_ batteries. (**A**) CV plots of CNTs and pFe^II^Pc cathode in the Ar and CO_2_ atmosphere. (**B**) Full deep discharge/charge profiles of CNTs and solid pFe^II^Pc cathode under Ar and CO_2_ at a current density of 100 mA g^−1^. mAh, mA·hour. (**C**) Comparison of the galvanostatic charge-discharge profiles of Li-CO_2_ batteries with pFe^II^Pc and without pFe^II^Pc within the cutoff capacity of 1000 mA·hour g^−1^. (**D**) Rate performance of Li-CO_2_ batteries with and without pFe^II^Pc. (**E**) Potential gap comparison between pFe^II^Pc and the CNT cathode at different current densities. (**F**) Recyclability of the Li-CO_2_ battery with pFe^II^Pc with a cutoff capacity of 1000 mA·hour g^−1^ at 100 mA g^−1^. (**G**) Median discharge/charge voltage plateau voltages for pFe^II^Pc and CNT electrode at 100 mA g^−1^, with the inset purple dots referring to energy efficiency. (**H**) Charge-discharge profiles of the Li-CO_2_ battery with pFe^II^Pc as solid-state redox mediators across a wide temperature range with a cutoff capacity of 500 mA·hour g^−1^ at 20 mA g^−1^.

pFe^II^Pc, cobalt polyphthalocyanine, nickel polyphthalocyanine, and copper polyphthalocyanine were used as a catalytic cathode in Li-CO_2_ batteries. The Li-CO_2_ battery using solely pFe^II^Pc as the solid-state redox mediator displays a discharge voltage plateau of 3.2 V (fig. S7). Li-CO_2_ batteries were assembled with and without pFe^II^Pc to further investigate the electrochemical performance of the solid-state redox mediator pFe^II^Pc. The full charge-discharge process was conducted within a voltage range of 2.0 to 4.5 V at 100 mA g^−1^, as provided in Fig. 2B. Figure 2B shows that the solid pFe^II^Pc cathode in the CO_2_ atmosphere exhibits outstanding stability, with an initial discharge capacity as high as 28,790 mA·hour g^−1^ and a reversible capacity of 26,338 mA·hour g^−1^. Also, the solid pFe^II^Pc cathode shows severely limited performance in the Ar atmosphere, with discharge and charge capacities of only 1560 and 1364 mA·hour g^−1^, respectively. In the control group, the Li-CO_2_ battery without pFe^II^Pc demonstrates limited performance under CO_2_, achieving merely 9468 mA·hour g^−1^ discharge capacity and 7531 mA·hour g^−1^ recharge capacity. As illustrated in Fig. 2C, the Li-CO_2_ battery containing pFe^II^Pc demonstrates not only a high discharge voltage plateau of 3.2 V (considerably exceeding the theoretical discharge voltage of 3.01 V) but also achieves a reduced charge voltage of 3.7 V compared to its pFe^II^Pc-free counterpart. It exhibits a substantially lower overpotential of merely 0.58 V, representing just one-third of that observed in the control group (1.74 V). Meanwhile, rate capability is a critical parameter for evaluating reaction kinetics, which was assessed at multiple current densities under a fixed cutoff capacity of 1000 mA·hour g^−1^. As shown in Fig. 2 (D and E), with the current density increased progressively from 100 to 1000 mA g^−1^, the Li-CO_2_ battery with pFe^II^Pc demonstrated a controlled voltage gap expansion (0.59 → 1.32 V), markedly lower than the pronounced polarization increase (1.66 → 2.08 V) observed in the pFe^II^Pc-free counterpart cell. Notably, upon recovering the current density to 100 mA g^−1^, the potential gap of the Li-CO_2_ battery with pFe^II^Pc returned to 0.73 V. The Li-CO_2_ battery with pFe^II^Pc maintains a discharge voltage plateau exceeding 3.01 V even at 600 mA g^−1^, with a high median discharge voltage of 2.8 V persisting at ultrahigh 1000 mA g^−1^. To systematically evaluate the cycling stability, Li-CO_2_ batteries with and without pFe^II^Pc were subjected to extended cycling tests at varying current densities with a cutoff capacity of 1000 mA·hour g^−1^. Figure 2F demonstrates the stable cycling stability of the pFe^II^Pc cathode, maintaining stable operation for 40 cycles with a 3.01-V discharge termination potential and <4.1-V charge voltage plateau, and the energy efficiency stays above 75%. Nevertheless, the accumulation of discharge products and partial deactivation of the catalyst during prolonged cycling bring about a decay in the discharge plateau of the Li-CO_2_ battery after 45 cycles (fig. S8). Moreover, the Li-CO_2_ battery with pFe^II^Pc also displays prolonged cycling stability, maintaining continuous operation for 1482 hours at 100 mA g^−1^ (Fig. 2G and fig. S9). This is due to the extremely low solubility of pFe^II^Pc in the electrolyte, as well as the fact that during cycling in the Li-CO_2_ battery, the crystal structure of pFe^II^Pc remains largely intact without significant evolution or degradation, and the valence state of its catalytic active sites remains unchanged (figs. S10 and S11). To evaluate high-rate cycling stability, Li-CO_2_ batteries with pFe^II^Pc were tested at elevated current densities under a cutoff capacity of 1000 mA·hour g^−1^. The cells exhibited sustained operation for 191 and 139 hours at 600 and 1000 mA g^−1^ (fig. S12). This special electrochemical performance stems directly from pFe^II^Pc’s superior conductive and catalytic activity, which mitigates polarization and enhances reaction kinetics.

The Li-CO_2_ battery with pFe^II^Pc maintains excellent performance across an ultrawide operating temperature range beyond room temperature. The electrochemical performance of the Li-CO_2_ battery with pFe^II^Pc under a cut-off capacity of 500 mA·hour g^−1^ was evaluated at temperatures ranging from −40° to 80°C (−40°, −30°, −20°, 0°, 20°, 40°, 60°, and 80°C), as presented in Fig. 2H. Despite the fact that the voltage gap is slightly larger at −30° and −20°C, the pFe^II^Pc-based Li-CO_2_ battery retains a stable 2.9-V discharge voltage plateau and consistently operates above 3.01 V from 0° to 80°C (fig. S13). The Li-CO_2_ battery with pFe^II^Pc demonstrates excellent electrochemical performance over a wide temperature range. However, the discharge plateau inevitably declines with decreasing temperature—a phenomenon rooted in the temperature-dependent electrical conductivity of pFe^II^Pc, which diminishes as the temperature falls. Furthermore, theoretical calculations also reveal that the Gibbs free energy barrier of the rate-determining step during the discharge process of Li-CO_2_ batteries rises with decreasing temperature (figs. S14 and S15). This further corroborates that kinetic limitation is another critical factor underlying the voltage decline. The battery maintains normal operation even at −40°C, which is attributed to the extraordinary catalytic properties of pFe^II^Pc across an ultrawide operating temperature range. At the same time, the porous structure in the separator also helps to lower the freezing point of the electrolyte to some extent, further enhancing the performance of the Li-CO_2_ battery at low temperatures (fig. S16). In situ temperature-dependent Raman spectroscopy further confirms this observation (Fig. 3, A and B, and fig. S17), with pFe^II^Pc maintaining structural integrity over −60° to 80°C, confirming superior wide-temperature stability. In addition, the Li-CO_2_ battery using pFe^II^Pc as the solid-state redox mediator exhibits stable cycling performance at −20° and 80°C (fig. S18).

**Fig. 3.**
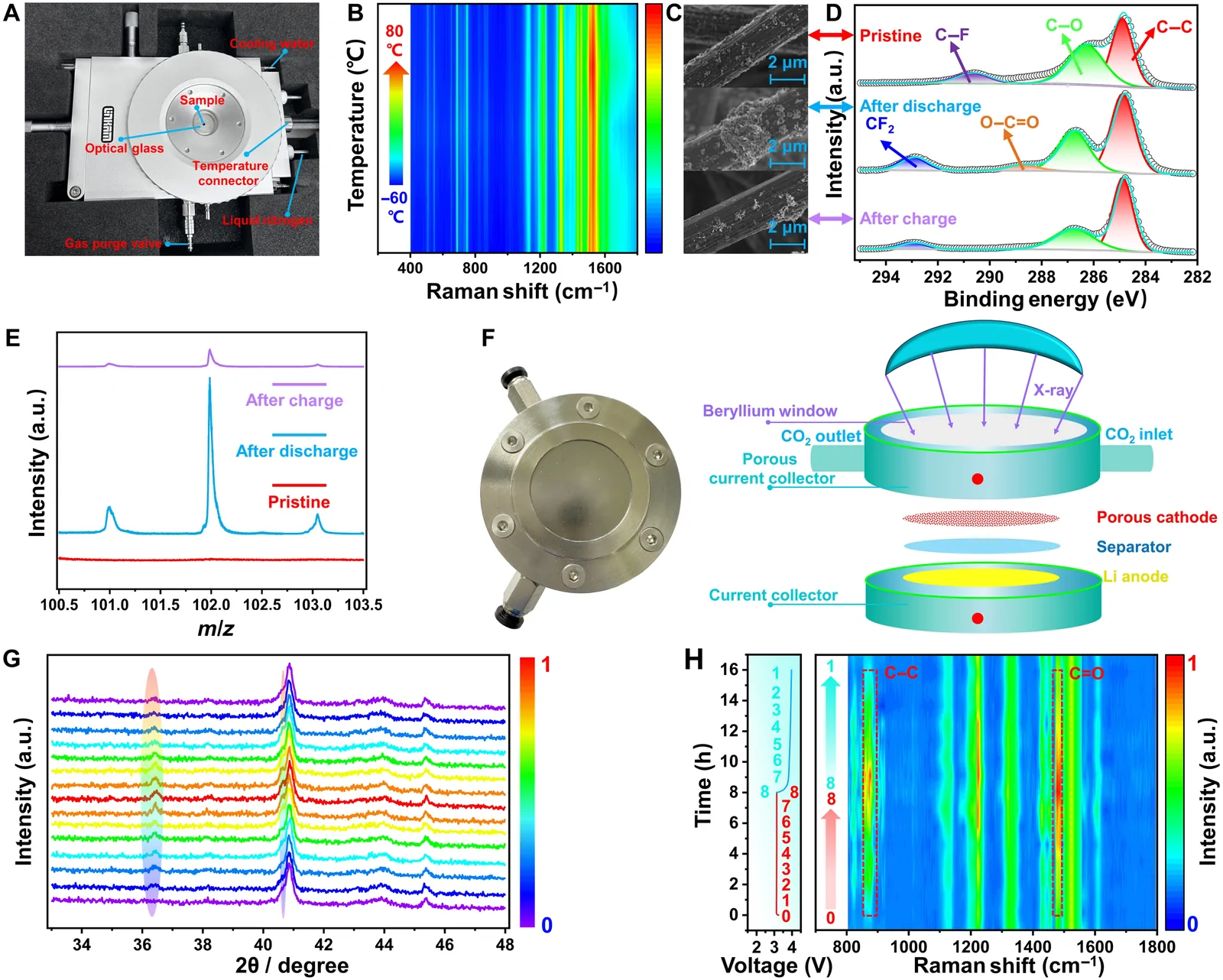
Multicharacterization analysis of discharge products. (**A**) Optical image of the variable-temperature in situ Raman device. (**B**) In situ Raman spectra of pFe^II^Pc measured at different temperatures. (**C**) SEM of Li-CO_2_ batteries with pFe^II^Pc as solid-state redox mediators at pristine, discharged, and recharged states, respectively. (**D**) C 1s XPS spectra of Li-CO_2_ batteries with pFe^II^Pc as solid-state redox mediators at pristine, discharged, and recharged states. a.u., arbitrary units. (**E**) *m*/*z* 100.5 to 103.5. (**F**) Structure of in situ Li-CO_2_ XRD battery. (**G**) In situ XRD patterns of Li-CO_2_ batteries with pFe^II^Pc at 200 mA g^−1^. (**H**) Charge-discharge profiles of Li-CO_2_ batteries with pFe^II^Pc at 200 mA g^−1^ with 16 selected states and the corresponding in situ Raman spectra. h, hours.

### Product characterization of Li-CO_2_ batteries catalyzed by pFe^II^Pc

To investigate the discharge products and the reversibility of the Li-CO_2_ battery, a series of in situ and ex situ characterizations were conducted during the charge-discharge process. As evident in Fig. 3C, products deposited on the carbon fibers were clearly visible after discharging but disappeared following complete recharge. Ex situ x-ray photoelectron spectroscopy (XPS) and time-of-flight secondary ion mass spectrometry (TOF-SIMS) were performed to further identify the species of the discharge products. The XPS spectra of C 1s and Li 1s were comparatively analyzed in the pristine state, after discharge, and after recharge (Fig. 3D and fig. S19). The XPS spectra of C 1s from the discharged electrode clearly exhibits characteristic peaks at 288.90 eV (O─C═O), which unambiguously confirm the formation of Li_2_C_2_O_4_ as the discharge product.

Two prominent signals observed after discharge at mass/charge ratios (*m*/*z*) of 101.98 and 88 in Fig. 3E and fig. S20 exhibit exact correspondence with Li_2_C_2_O_4_ and disappear after charging, providing direct evidence for Li_2_C_2_O_4_ formation during discharge. In addition, the TOF-SIMS results show that no prominent signals appear at *m*/*z* 23 and 60, confirming that Li_2_CO_3_ is absent in the products (fig. S20). The ex situ x-ray diffraction (XRD) and Raman analyses of the deep discharge products also confirm that Li_2_C_2_O_4_ is the final discharge product (fig. S21). The dynamic evolution of discharge/charge processes was monitored in real time using specially designed in situ XRD and Raman electrochemical cells (Fig. 3F and fig. S22). Figure 3G presents the galvanostatic charge-discharge profiles of the in situ Li-CO_2_ XRD battery using the pFe^II^Pc cathode, demonstrating a reversible capacity of 1600 mA·hour g^−1^ at a current density of 200 mA g^−1^. The XRD diffraction peak intensities of Li_2_C_2_O_4_ at 2θ = 36.44° and 40.64° progressively intensify with increasing discharge depth and systematically diminish during the charging process, exhibiting a reversible evolution pattern. Consistent with the in situ XRD findings, complementary in situ Raman spectroscopy corroborated these observations. As Fig. 3H documents, the Raman peak intensities associated with Li_2_C_2_O_4_ at 865 cm^−1^ (C─C) and 1486 cm^−1^ (C═O) exhibited a discharge depth–dependent enhancement. These spectral features exhibited progressive attenuation during charging and ultimately disappeared upon reaching the full state of charge. Furthermore, the discharge product of the Li-CO_2_ battery without pFe^II^Pc as the solid-state redox mediator is Li_2_CO_3_ (fig. S23). pFe^II^Pc as the solid-state redox mediator in the Li-CO_2_ battery that not only directs the discharge product to Li_2_C_2_O_4_ but also promotes reversible CO_2_ redox.

### Mechanistic analysis of the ultrahigh discharge plateau

Quantitative differential electrochemical mass spectrometry (DEMS) was performed to analyze the electrons transferred on the pFe^II^Pc surface as well as the consumption and evolution of CO_2_. Dual-gas-path in situ DEMS testing during the charge-discharge process is illustrated in Fig. 4A and fig. S24. The transferred charge-to-CO_2_ consumption ratio (e^−^/CO_2_) for the reaction 2Li^+^ + 2CO_2_ + 2e^−^ → Li_2_C_2_O_4_ is determined to be 1 during the charge-discharge process. The near-unity e^−^/CO_2_ ratio (0.97) observed during discharge suggests highly efficient CO_2_ utilization and effective Li_2_C_2_O_4_ formation on the pFe^II^Pc electrode (Fig. 4B). Moreover, analyses of the evolved gas species and the CO_2_ evolution mechanism were conducted during the charge process. As illustrated in Fig. 4C and fig. S25, only CO_2_ is generated during charging, with no detectable generation of O_2_ and CO, indicating the reversibility of CO_2_ evolution. DEMS measurements show a 1.04 e^−^/CO_2_ ratio during charging, consistent with the theoretical value of 1 e^−^/CO_2_ predicted by the reversible CO_2_ evolution reaction (Li_2_C_2_O_4_ → 2Li^+^ + 2CO_2_ + 2e^−^). Variations in charge/discharge currents and carrier gas flow rates result in differing CO_2_ fluxes, yet the experimental-to-theoretical value ratio is close to 1 for both processes. Therefore, these findings confirm that the Li-CO_2_ battery using pFe^II^Pc as the solid-state redox mediator undergoes a two-electron transfer process, with Li_2_C_2_O_4_ as the main discharge product.

**Fig. 4.**
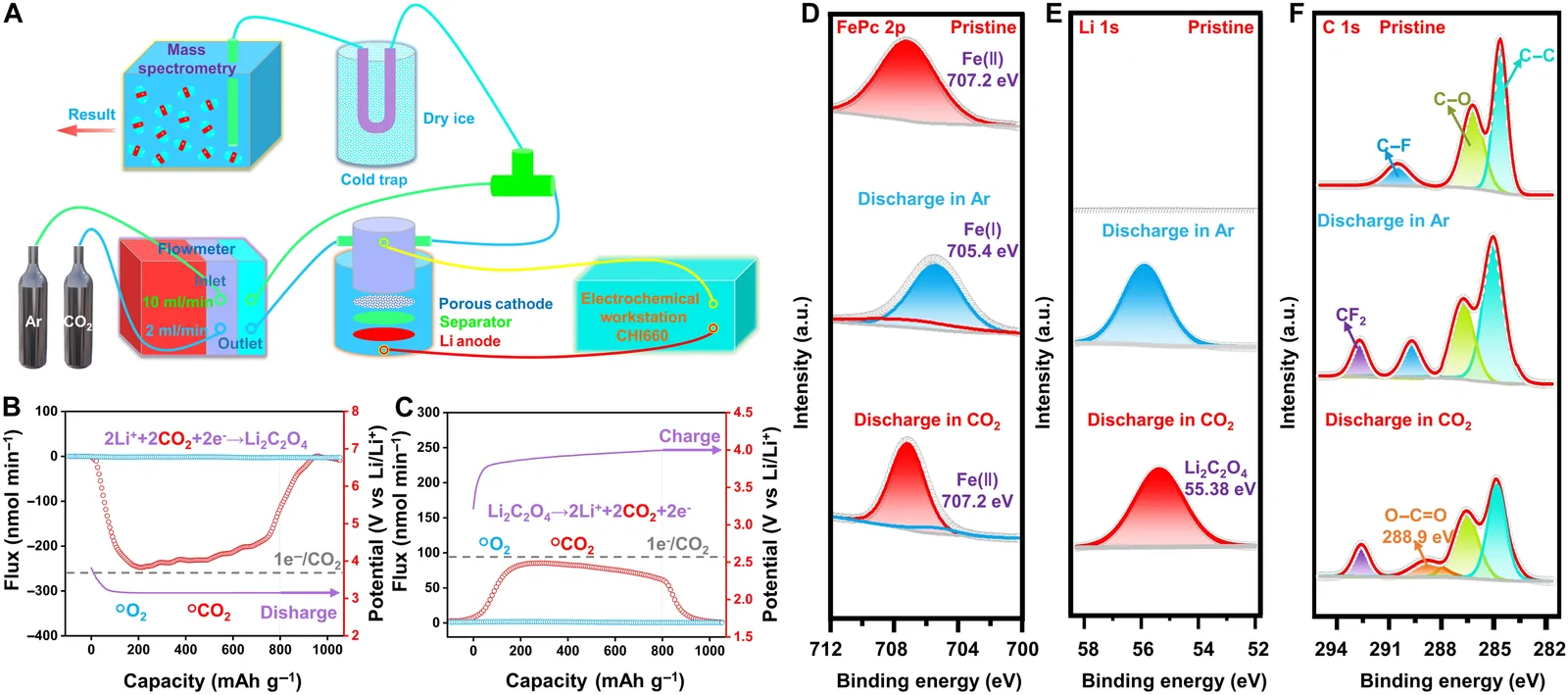
Mechanism analysis on the high discharge plateau of the Li-CO_2_ battery. (**A**) Schematic diagram of the in-situ DEMS for the discharge process of the Li-CO_2_ battery. In situ DEMS profiles of Li-CO_2_ batteries with pFe^II^Pc as solid-state redox mediators during (**B**) the discharge process and (**C**) charge process. (**D**) XPS pFe^II^Pc 2p spectra and (**E**) Li 1s and (**F**) C 1s of the solid-state pFe^II^Pc cathode at different atmospheres and discharge stages.

To elucidate the formation mechanism of Li_2_C_2_O_4_ and ultrahigh discharge platform mechanism on the solid pFe^II^Pc cathode, the cathode surface composition at various electrochemical stages was systematically analyzed by ex situ XPS and density functional theory (DFT). First, XPS analysis reveals that the Fe 2p spectrum of the pristine pFe^II^Pc cathode under the Ar atmosphere (Fig. 4D, top) exhibits a single characteristic peak at ∼707.2 eV, confirming the exclusive presence of Fe(II) in the pristine pFe^II^Pc cathode. As the discharge progresses (Fig. 4D, middle), a new peak emerges at ∼705.4 eV under Ar, attributed to the increased Fe(I) state. Concurrently, the Fe(I) signal diminishes substantially, indicating near-complete reduction to Fe(I). Notably, the Fe(I) valence state shows no change after CO_2_-assisted discharge, in sharp contrast to the Ar case (Fig. 4D, bottom). This provides direct evidence for the rapid electron shuttle mechanism: Reduced Fe(I) promptly donates its electrons to CO_2_ and is therefore oxidized back to Fe(II). This conclusion is fully consistent with the CV results, corroborating the proposed reaction mechanism and ultrahigh discharge platform mechanism. Subsequently, the formation of Li_2_C_2_O_4_ in the CO_2_ atmosphere is confirmed by the appearance of two characteristic peaks: C 1s (O─C═O) at 288.90 eV and Li 1s at 55.34 eV (Fig. 4, E and F). This phenomenon was not observed in the Ar atmosphere, confirming the CO_2_-dependent nature of Li_2_C_2_O_4_ formation and pFe^II^Pc solely as a redox mediator. An unexpected Li signal appeared on the electrode after discharge in Ar, which is attributed to the precipitation of lithium salts in the electrolyte and the contribution of Li from the cathode electrolyte interstate. Furthermore, DFT calculations reveal that pFe^II^Pc exhibits an electron affinity of 4.91 eV, accompanied by electron cloud redistribution upon CO_2_ adsorption on pFe^I^Pc. (figs. S26 and S27). This thermodynamic preference ensures pFe^II^Pc as the preferential electron acceptor: pFe^II^Pc is preferentially reduced (pFe^II^Pc + e^−^ → pFe^I^Pc) and subsequently mediates electron donation to CO_2_ for the ensuing electrochemical reactions. In addition, a control system with Fe(III) active sites was constructed, demonstrating that uncoordinated Fe(III) lacking phthalocyanine ligands fails to catalyze the reduction reaction efficiently, whereas the present system realizes efficient electrocatalysis via the Fe(II)/Fe(I) redox cycle (fig. S28).

### Reaction pathway for Li_2_C_2_O_4_ formation from CO_2_ catalyzed by pFe^II^Pc

The DFT calculation was performed to systematically investigate the stability of Li_2_C_2_O_4_ on the pFe^II^Pc surface. Figure 5A and figs. S29 to S31 present the DFT-optimized adsorption configurations and corresponding adsorption energies of Li_2_C_2_O_4_ on C_32_H_16_N_8_Fe, C_58_H_26_N_16_Fe_2_, and carbon nanotubes (CNTs). The calculated adsorption energy of Li_2_C_2_O_4_ on C_32_H_16_N_8_Fe (−1.21 eV) is markedly higher than those on C_58_H_26_N_16_Fe_2_ (−0.68 eV) and CNTs (−0.61 eV), indicating preferential stabilization of Li_2_C_2_O_4_ as discharge products on the C_32_H_16_N_8_Fe surface during the discharge process. To clarify the reaction mechanism of Li_2_C_2_O_4_ on the redox mediator surface during discharge, two distinct reaction pathways using monomers C_32_H_16_N_8_Fe and C_58_H_26_N_16_Fe_2_ as catalytic units were computationally investigated. Figure 5B presents the free energy profiles for the most probable 2e^−^/2CO_2_ nucleation pathways of Li_2_C_2_O_4_ formation on surfaces C_32_H_16_N_8_Fe and C_58_H_26_N_16_Fe_2_. The two dominant reaction pathways proceed as follows:

**Fig. 5.**
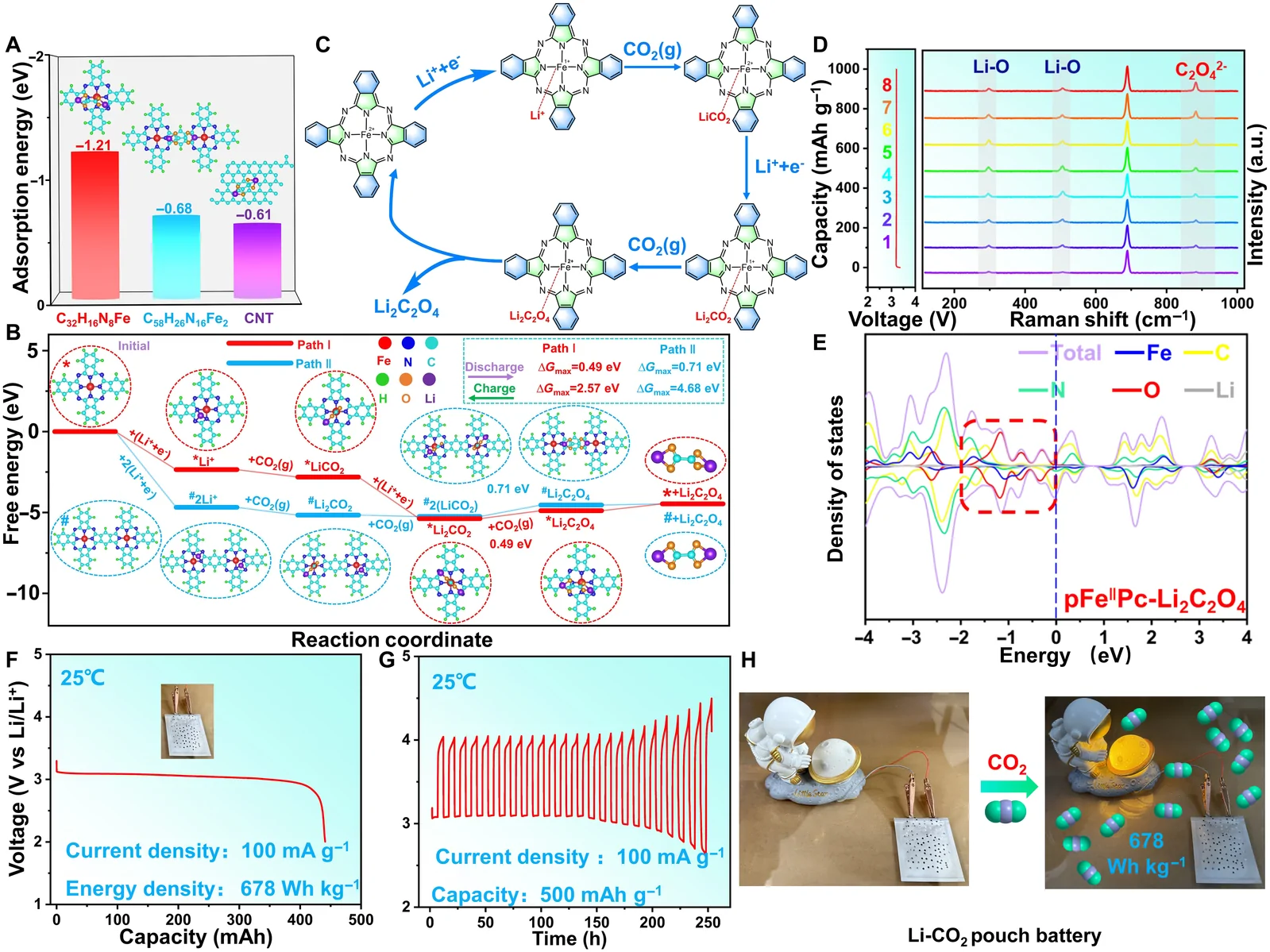
Theoretical calculation and practical application verification. (**A**) Optimized structure and adsorption energy of Li_2_C_2_O_4_ on C_32_H_16_N_8_Fe, C_58_H_26_N_16_Fe_2_, and CNTs. (**B**) Free energy diagrams of different nucleation pathways of Li_2_C_2_O_4_ on C_32_H_16_N_8_Fe and C_58_H_26_N_16_Fe_2_. (**C**) Proposed mechanism of pFe^II^Pc as solid-state redox mediators that mediated the CO_2_ reduction process in the Li-CO_2_ battery. (**D**) Discharge profiles of Li-CO_2_ batteries with pFe^II^Pc with eight selected states and the corresponding in situ Raman spectra. (**E**) Fermi level of C_32_H_16_N_8_Fe after Li_2_C_2_O_4_ adsorption. (**F**) Full discharge capacity of a Li-CO_2_ pouch cell fabricated with the pFe^II^Pc cathode. (**G**) Cycling performance of a Li-CO_2_ pouch cell at room temperature. (**H**) The Li-CO_2_ pouch cell with pFe^II^Pc lights up the LED of the astronaut model.

Path I

$$\begin{array}{c} {}^{*}+\left({\text{Li}}^{+}+e^{-}\right)\to {}^{*}{\text{Li}}^{+}+{\text{CO}}_{2}\left(g\right)\to {}^{*}{\text{LiCO}}_{2}+\left({\text{Li}}^{+}+e^{-}\right)\to {}^{*}{\text{Li}}_{2}{\text{CO}}_{2}\\ +{\text{CO}}_{2}\left(g\right)\to {}^{*}{\text{Li}}_{2}C_{2}O_{4}\to {}^{*}+{\text{Li}}_{2}C_{2}O_{4} \end{array}$$

Path II

$$\begin{array}{c} {}^{\#}+2\left({\text{Li}}^{+}+e^{-}\right)\to {\;}^{\#}{\text{2Li}}^{+}+{\text{CO}}_{2}\left(g\right)\to {\;}^{\#}{\text{LiCO}}_{2}{\text{Li}}^{+}+{\text{CO}}_{2}\left(g\right)\\ \to {\;}^{\#}2\left({\text{LiCO}}_{2}\right)\to {\;}^{\#}{\text{Li}}_{2}C_{2}O_{4}\to {\;}^{\#}+{\text{Li}}_{2}C_{2}O_{4} \end{array}$$

In both pathways, all Li^+^-involving steps with electron transfer exhibit exothermic downhill profiles in the free energy diagrams, confirming their spontaneous nature. However, both CO_2_ adsorption on *Li_2_CO_2_ to form *Li_2_C_2_O_4_ in pathway I and that on ^#^2(LiCO_2_) to form ^#^Li_2_C_2_O_4_ in pathway II exhibit endothermic characteristics. These endothermic uphill steps serve as the rate-determining steps for Li_2_C_2_O_4_ formation in both pathways I and II. The activation energy barrier for the rate-determining step in pathway I (0.49 eV) is substantially lower than that in pathway II (0.71 eV). The overall Gibbs free energy change (∆*G*) for Li_2_C_2_O_4_ decomposition in pathway I (2.57 eV) is considerably more negative than that in pathway II (4.68 eV). These findings unambiguously demonstrate that the steady Li_2_C_2_O_4_ electrochemistry via the 2e^−^/2CO_2_ reaction preferentially follows pathway I in pFe^II^Pc-based Li-CO_2_ batteries (Fig. 5C). On the basis of the optimal reaction pathway determined through dynamic simulations via Li_2_C_2_O_4_ formation, we have captured the dynamic evolution of characteristic vibrational peaks of Li-O and C_2_O_4_^2−^ intermediates during discharge in in situ Raman spectroscopy (Fig. 5D). Li_2_C_2_O_4_ can serve as the final discharge product in the Li-CO_2_ battery because of its stronger adsorption energy with pFe^II^Pc. In addition, projected density of states analysis shows that the interaction between Li_2_C_2_O_4_ and pFe^II^Pc primarily occurs near the Fermi level (−2 to 0 eV). In comparison to Li_2_CO_3_ (−4 to 0 eV), the O-p orbital distribution in *Li_2_C_2_O_4_ is narrower, indicating that the p-electrons of the oxygen atoms in *Li_2_C_2_O_4_ are more concentrated near the Fermi level, resulting in a lower and more stable energy state (Fig. 5E and figs. S32 and S33).

Proof-of-concept Li-CO_2_ pouch cells were demonstrated using pFe^II^Pc as the solid-state redox mediator paired with lithium bis(trifluoromethanesulfonyl) imide/tetraglyme (LiTFSI/TEGDME) electrolytes. As shown in Fig. 5F, the Li-CO_2_ pouch cell (5.5 cm by 4 cm, 2.19 g) exhibits a discharge voltage plateau of 3.1 V with a high discharge capacity of 479 mA·hour at room temperature under 100 mA g^−1^, delivering an impressive energy density of 678 W·hour kg^−1^ based on the total pouch cell mass. In addition, the Li-CO_2_ pouch cell using pFe^II^Pc as solid-state redox mediator cathodes with a cutoff capacity of 500 mA·hour g^−1^ at 100 mA g^−1^ maintains a discharge voltage plateau above 3.01 V for 19 reversible cycles, with a total cycle duration of up to 251 hours (Fig. 5G). As displayed in Fig. 5H, the pouch cell successfully powered light-emitting diodes (LEDs) under simulated Martian conditions (95% CO_2_), demonstrating that the pFe^II^Pc-based Li-CO_2_ battery technology holds significant potential for practical applications.

## DISCUSSION

In summary, we proposed a novel strategy that uses pFe^II^Pc with ultrahigh electron affinity as the solid-state redox mediator to achieve the Li_2_C_2_O_4_-product pathway while elevating the discharge voltage plateau of Li-CO_2_ batteries to 3.2 V, enabling long-term stable reversible cycling on the basis of the Li_2_C_2_O_4_ pathway. The reaction mechanism of Li-CO_2_ batteries using pFe^II^Pc as the cathode material is collectively elucidated by electrochemical studies, in situ spectroscopic characterization, and DFT calculation. During discharge, pFe^II^Pc preferentially accepts electrons from the anode instead of CO_2_, being reduced to pFe^I^Pc. Subsequently, pFe^I^Pc rapidly transfers these electrons to CO_2_ (thereby being oxidized back to pFe^II^Pc), and ultimately, the reduced CO_2_ reacts with Li^+^ to form Li_2_C_2_O_4_. Notably, pFe^II^Pc exhibits strong adsorption energy toward Li_2_C_2_O_4_, thereby stabilizing the Li_2_C_2_O_4_ product. Given that the reduction potential of pFe^II^Pc accepting electrons to form pFe^I^Pc exceeds 3.01 V, this process results in the battery demonstrating a discharge voltage plateau higher than its theoretical value. As a proof-of-concept reliability verification candidate, pFe^II^Pc demonstrated exceptional catalytic kinetics in the Li-CO_2_ battery, delivering an ultrahigh discharge capacity of 28,790 mA·hour g^−1^ and reversible cyclability exceeding 1400 hours at 100 mA g^−1^. The introduction of pFe^II^Pc not only enables the Li_2_C_2_O_4_ pathway in Li-CO_2_ batteries but also achieves a discharge voltage plateau of 3.2 V. The designed Li-CO_2_ battery system enables superb wide-temperature operability (−40° to 80°C). Ultimately, our engineered pouch cell achieves an energy density of 678 W·hour kg^−1^ while maintaining stable cycling performance over 251 hours. This research provides a universally effective strategy for the practical application of Li-CO_2_ batteries.

## MATERIALS AND METHODS

### Materials

Super P, poly(vinylidene fluoride-*co*-hexafluoropropylene) [PVDF-HFP; average *M*_n_ (number-average molecular weight) of ∼130,000], and *N*-methyl-2-pyrrolidinone (NMP; 99.5%) were purchased from Sigma-Aldrich. The LiTFSI/TEGDME electrolyte (1.0 M) was purchased from Fosai. Carboxylated multiwalled CNTs (>95%) were purchased from MREDA. 1,2,4,5-Tetracyanobenzene was purchased from Sigma-Aldrich. Iron powder (Fe; 99.9%, 50 nm, 7439-89-6) was purchased from Macklin.

### Synthesis of pFe^II^Pc

1,2,4,5-Tetracyanobenzene (50 mg, 0.28 mmol) was mixed with iron powder (31.5 mg, 0.56 mmol). The mixture was thoroughly ground and transferred into a Pyrex tube measuring outer diameter × inside diameter = 10 mm by 8 mm. The Pyrex tube was then evacuated and flame sealed with the total length kept at ∼5.0 cm. The glass tube was heated to 400°C at 5°C/min and kept for 4 hours in a tube furnace under vacuum conditions. After cooling to room temperature, the product was washed several times with ethanol, iron chloride solution, and H_2_O and then dried at 60°C in a vacuum oven to afford a purple-black powder.

### Li-CO_2_ battery assembly

In the preparation of the porous cathode, for the experimental group, 45 wt % Super P, 45 wt % pFe^II^Pc, and 10 wt % PVDF were mixed in appropriate NMP. For the control group, 45 wt % Super P, 45 wt % CNTs, and 10 wt % PVDF were mixed in appropriate NMP. The resulting slurry was then pasted on a carbon paper, and the loading mass of active materials on the carbon cloth was ∼0.2 mg. The LiTFSI/TEGDME electrolyte (1.0 M) was used as the electrolyte. CR2032 coin batteries with holes for CO_2_ permeation were assembled with the as-prepared cathodes, glass fiber separator, Li metal anode, and 80 μl of as-prepared electrolyte in a glovebox filled with ultrapure argon. The assembled Li-CO_2_ batteries were then placed in a sealed glass bottle filled with CO_2_ and rested for 8 hours before testing with a Land battery tester at different temperatures.

### Characterizations and electrochemical measurements

CV testing was conducted in a specialized three-electrode cell, with a scan rate of 10 mV/s and a voltage testing range of 2.0 to 4.0 V during the process. The morphologies and structures of the discharge product were characterized by using scanning electron microscopy (SEM; Gemini SEM 500), and XRD patterns were collected with a Bruker D8 ADVANC diffractometer using Cu Kα radiation at a scan rate of 2° min^−1^, which was used to acquire the in situ XRD results. The Renishaw Raman spectrometer (InVia Qontor) with a 532-nm wavelength incident laser was used to obtain the in situ Raman spectra. XPS measurements were performed on a Thermo Fisher Scientific K-Alpha instrument. DEMS measurements were performed by a quadrupole mass spectrometer (PrismaPro) with a turbomolecular pump (Pfeiffer Vacuum). The mechanism analysis was obtained through TOF-SIMS (M6) and gas chromatography–mass spectrometry (Agilent 8890-5977C). A Land cycler (Wuhan Land Electronic Co., Ltd.) was used for galvanostatic charge-discharge tests of Li-CO_2_ batteries. The calculation of specific capacities and current densities were all based on the mass loading of pFePc and CNTs. The electrochemical stability of pFePc and CNTs were performed by CV conducted on an electrochemical workstation (CHI 660E) at the sweep rate of 1.0 mV s^−1^.

### Computational methods

Electronic calculations were performed to determine the adsorption energy and free energy profiles using spin-polarized DFT in the Vienna Ab initio Simulation Package (VASP) with a plane-wave basis set and the VASP method (*44*, *45*). The exchange-correlation potential was treated by using generalized gradient approximation with Perdew-Burke-Ernzerhof parametrization (*46*). A vacuum region of about 15 Å was applied to avoid the interaction between adjacent images. The energy cutoff was set to be 450 eV. A Monkhorst-Pack *k*-point mesh of 1 × 1 × 1 was used for structural optimizations. The structures were fully relaxed until the maximum force on each atom was less than 0.02 eV/Å, and the energy convergent standard was 10^−5^ eV.

Equation 2 is used to calculate the adsorption energy

$$E_{\text{ads}}=E_{\text{total}}-E_{\text{slab}}-E_{\text{adsorbate}}$$ (2)

where *E*_total_ is the total energy of the adsorbate-substrate system, *E*_slab_ is the energy of the clean substrate material, and *E*_adsorbate_ is the energy of the isolated adsorbate.

Equation 3 is used to calculate the free energy for each adsorbed and gas-state species. The Gibbs free energy *G* and change (Δ*G*) of each elementary step can be simplified to (*47*)

$$\text{∆}G=\text{∆}E+\text{∆}E_{\text{ZPE}}-T\text{∆}S+\text{∆}G_{\text{pH}}$$ (3)

In Eq. 3, *T* denotes the system temperature (298.15 K), while ∆*E*, ∆*E*_ZPE_, and ∆*S* represent the total electronic energy, zero-point energy, and entropy change of the reaction, respectively, as obtained from DFT calculations. ∆*G*_pH_ is the correction of pH value for Gibbs free energy, and pH is equal to zero in this work. The two dominant reaction pathways proceed as follows:

Pathway I:

i: C_32_H_16_N_8_Fe^II^ + Li^+^ + e^−^ → C_32_H_16_N_8_Fe^I^Li^+^

ii: C_32_H_16_N_8_Fe^I^Li^+^ + CO_2_(g) → C_32_H_16_N_8_Fe^II^LiCO_2_

iii: C_32_H_16_N_8_Fe^II^LiCO_2_ + Li^+^ + e^−^ → C_32_H_16_N_8_Fe^I^Li_2_CO_2_

iv: C_32_H_16_N_8_Fe^I^Li_2_CO_2_ + CO_2_(g) → C_32_H_16_N_8_Fe^II^Li_2_C_2_O_4_

v: C_32_H_16_N_8_Fe^II^Li_2_C_2_O_4_ → C_32_H_16_N_8_Fe^II^ + Li_2_C_2_O_4_

Pathway II:

i: C_58_H_26_N_16_Fe_2_^II^ + 2Li^+^ + 2e^−^ → C_58_H_26_N_16_Fe_2_^I^2Li^+^

ii: C_58_H_26_N_16_Fe_2_^I^2Li^+^ + CO_2_(g) → C_58_H_26_N_16_Fe^I^Fe^II^LiCO_2_Li^+^

iii: C_58_H_26_N_16_Fe^I^Fe^II^LiCO_2_Li^+^ + CO_2_(g) → C_58_H_26_N_16_Fe_2_^II^LiCO_2_CO_2_Li

iv: C_58_H_26_N_16_Fe_2_^II^LiCO_2_CO_2_Li → C_58_H_26_N_16_Fe_2_^II^Li_2_C_2_O_4_

v: C_58_H_26_N_16_Fe_2_^II^Li_2_C_2_O_4_ → C_58_H_26_N_16_Fe_2_^II^ + Li_2_C_2_O_4_
