# Supplementary material for: Ultrahigh discharge voltage plateau of 3.2 V achieved in Li-CO2 batteries using iron polyphthalocyanine
Source: Sci Adv. 2026 Oct 2;12(40):eaei5080. doi: 10.1126/sciadv.aei5080 (PMC13632758; doi:10.1126/sciadv.aei5080)
Supplement: Supplementary file 1 [file sciadv.aei5080_sm.pdf]

Science Advances
AAAS

Supplementary Materials for

Ultrahigh discharge voltage plateau of 3.2 V achieved in Li-CO_2 batteries using iron polyphthalocyanine

Ning Zhao *et al.*

Corresponding author: Zheng Meng, zhengmeng@ustc.edu.cn; Xiao-Fei Hu, xiaofei.hu@xjtu.edu.cn

Sci. Adv. **12**, eaei5080 (2026)
DOI: 10.1126/sciadv.aei5080

This PDF file includes:

Figs. S1 to S33

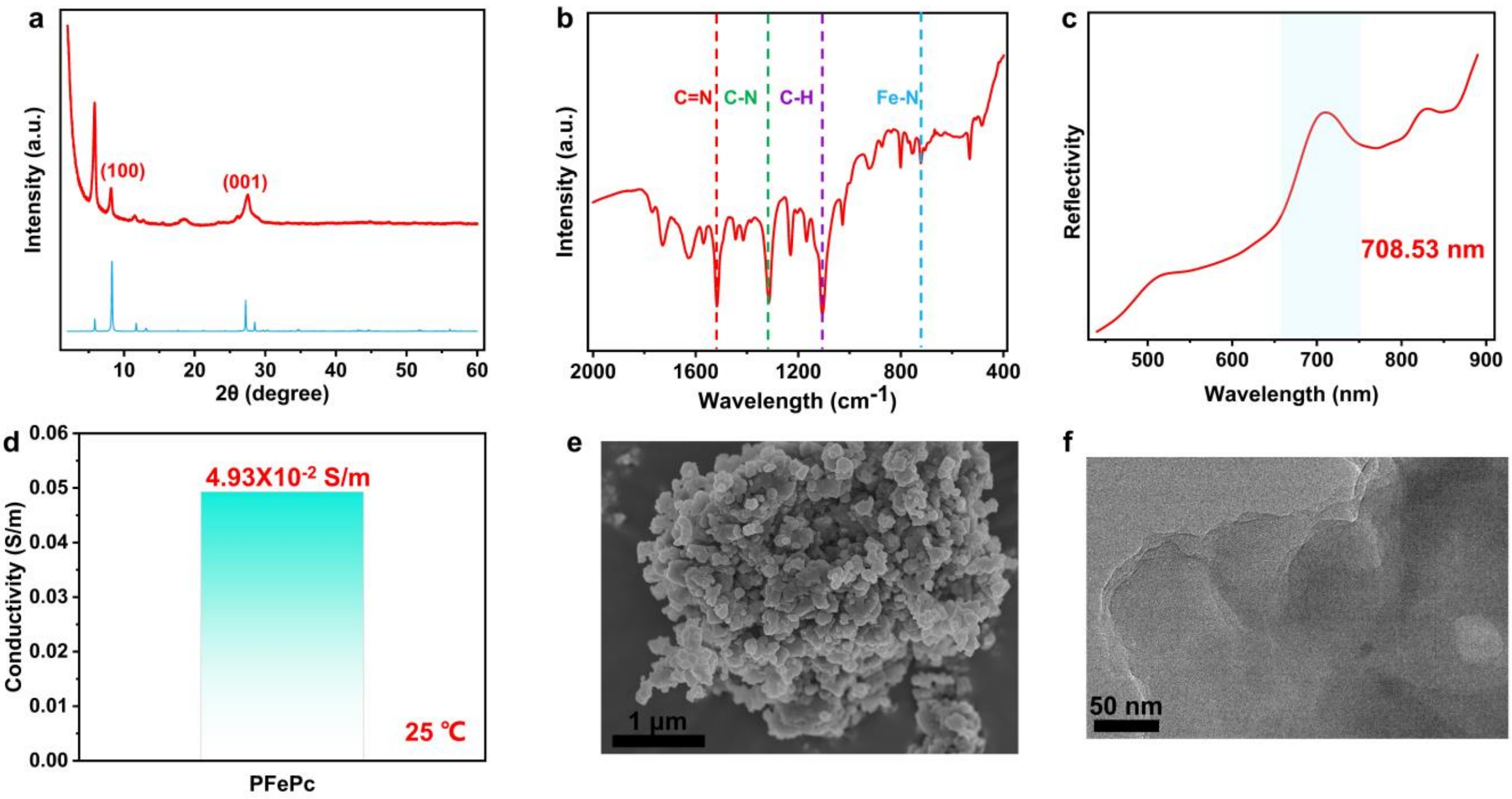

Fig. S1. Structural and property characterizations of pFe^{II}Pc. (a) XRD pattern (b) FT-IR spectrum (c) UV–vis spectrum (d) Conductivity (e) SEM and (f) TEM of solid-state redox mediator pFe^{II}Pc.

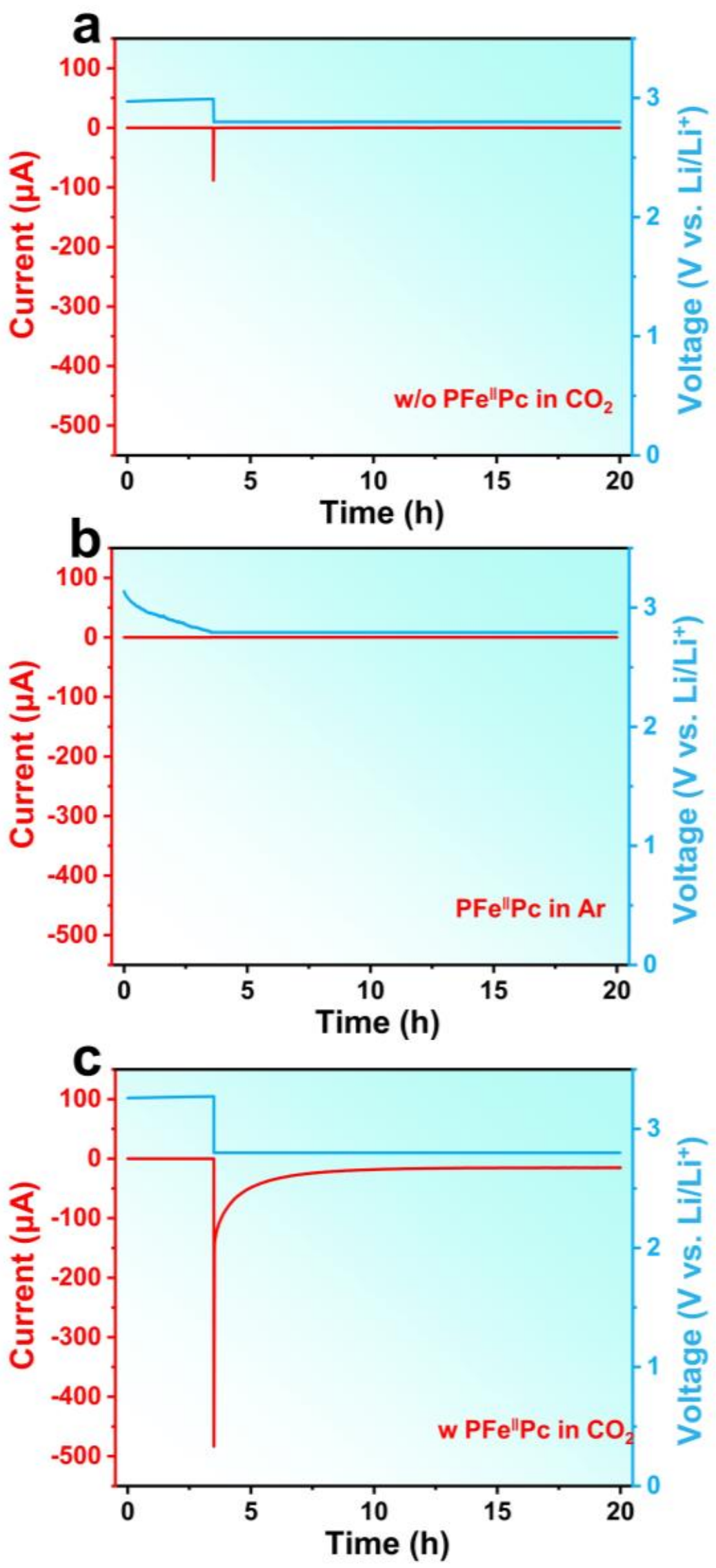

Fig. S2. Current-time test results. Current-Time curve under constant voltage of (a) CNTs and pFe^{II}Pc cathode in (b) Ar and (c) CO_2 atmosphere.

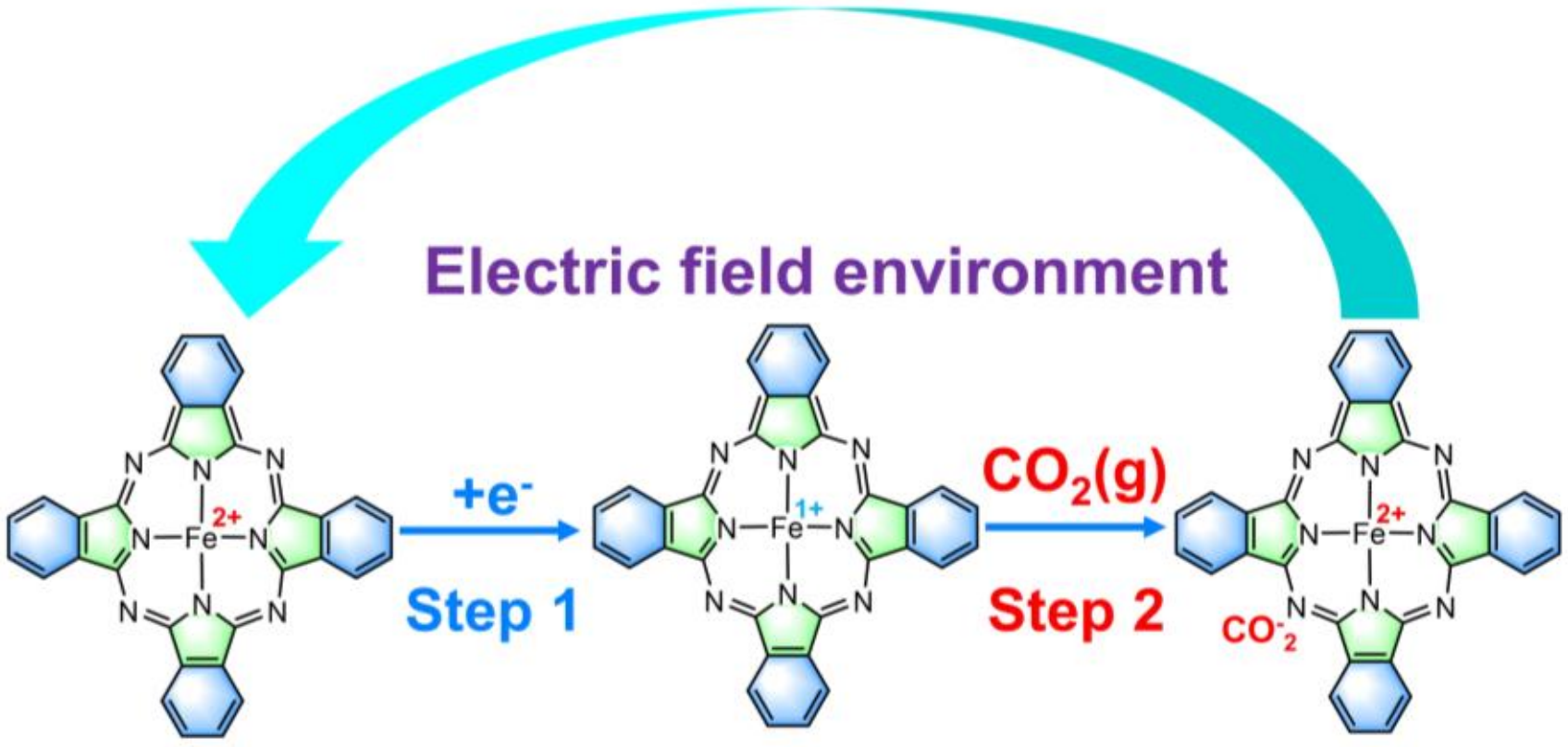

Fig. S3. Electrochemical reaction schematic. Schematic diagram of CO_2 reduction by pFe^{II}Pc via priority electron transfer.

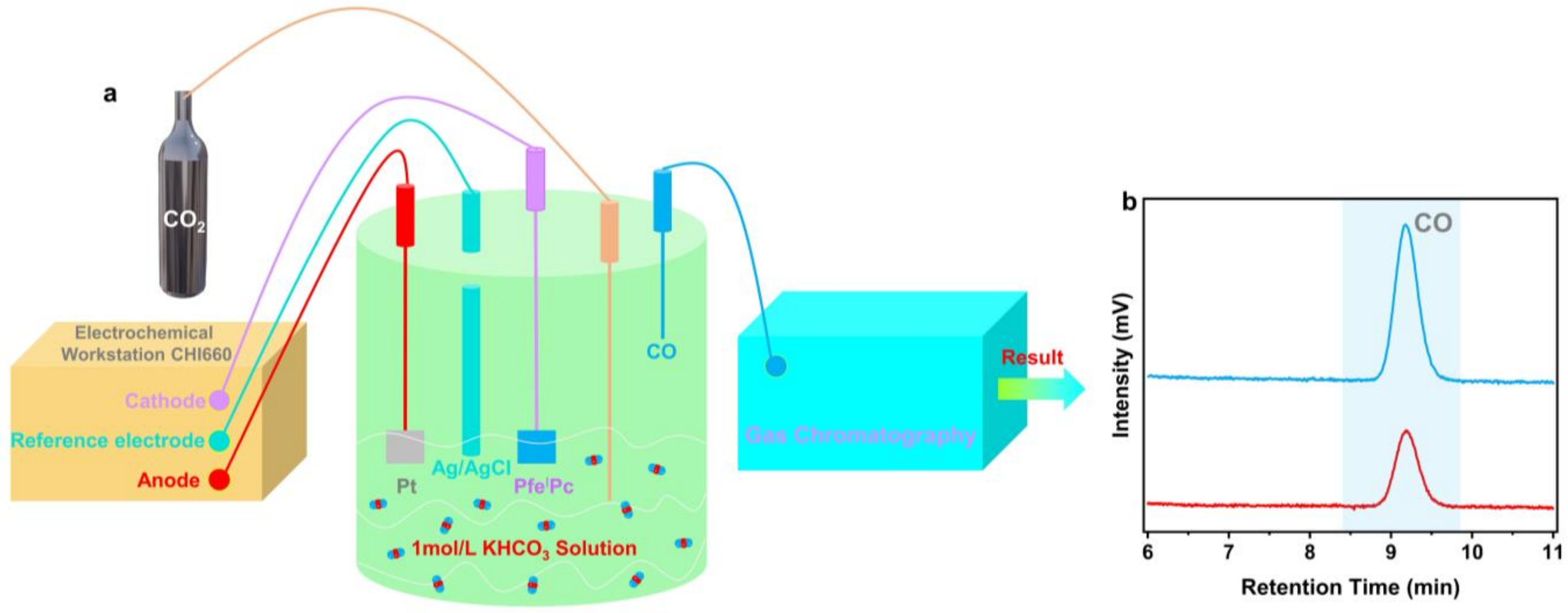

Fig. S4. Process and results of GC-MS characterizations. (a) Schematic diagram of in-situ online GC-MS. (b) GC-MS spectrum based on pFe^{I}Pc cathode during discharging in CO_2 atmosphere.

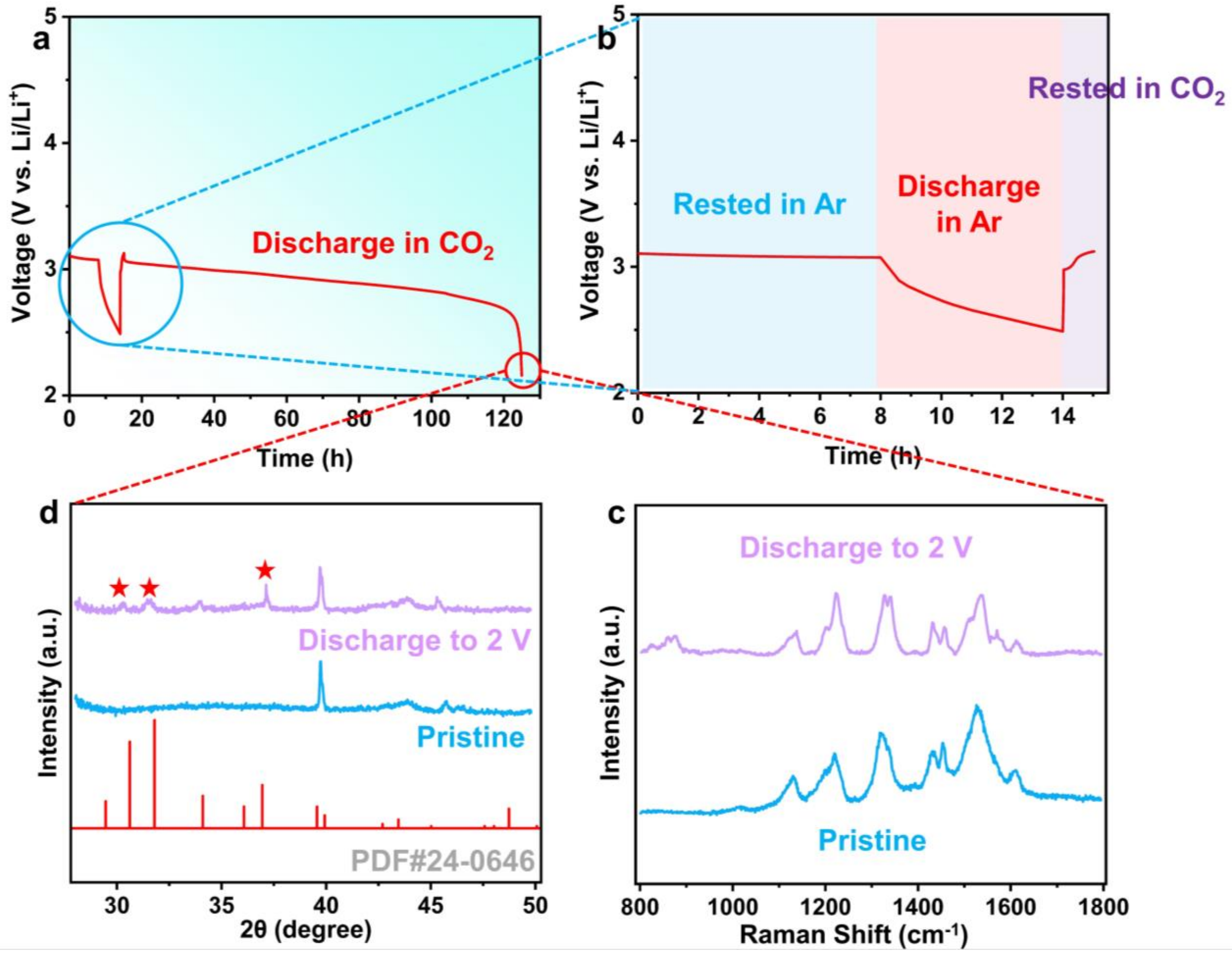

Fig. S5. Discharge profiles and XRD, Raman characterizations of Li-CO_2 batteries. (a) Discharge curve of Li-CO_2 battery using pFe^{II}Pc, first discharged in Ar and then in CO_2. (b) Magnified view of Fig. S5a. (c) XRD pattern and (d) Raman spectrum of discharge products.

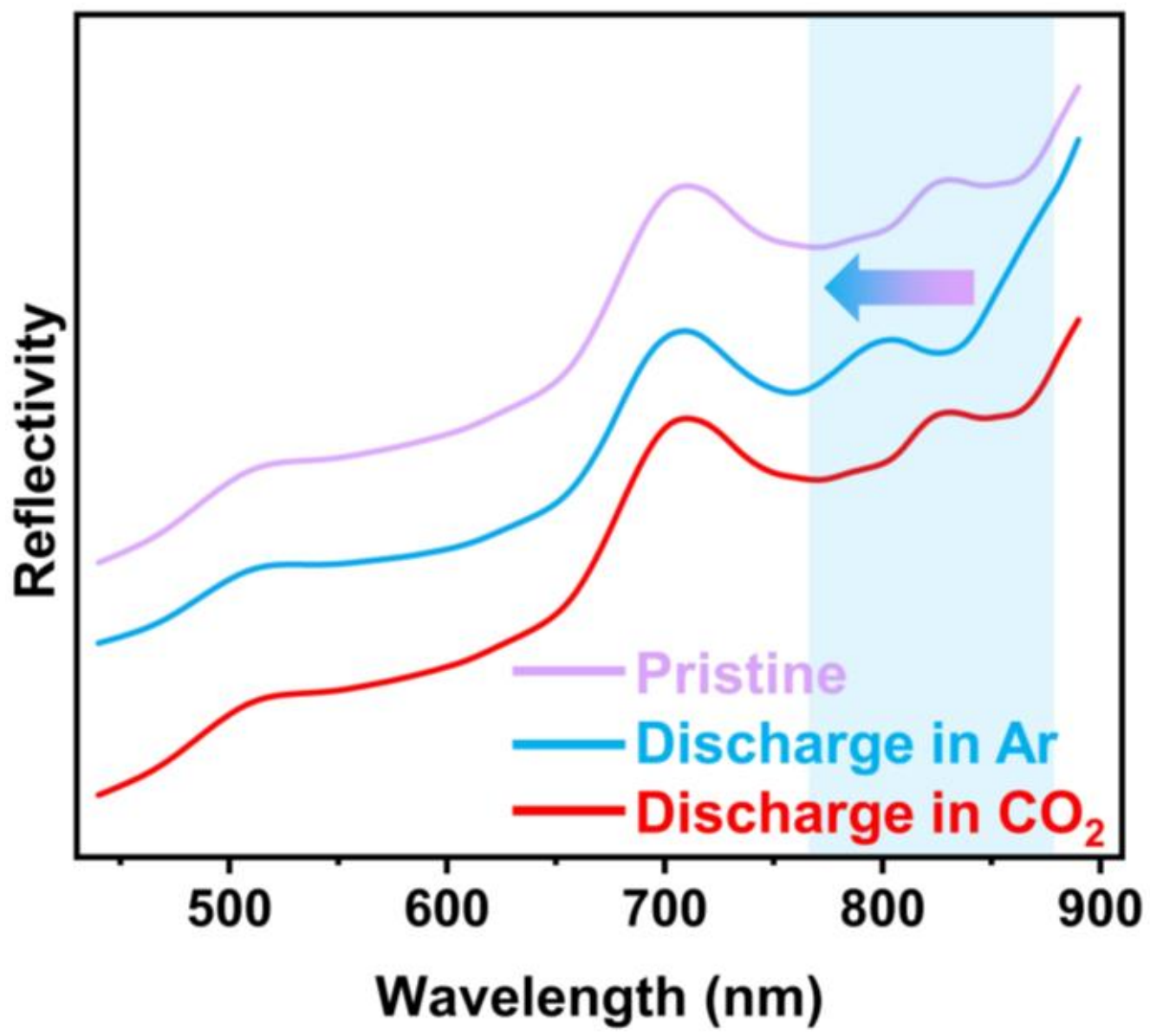

Fig. S6. UV-vis characterizations. UV-vis spectra of the pristine state, after discharge in an Ar and in a CO_2 atmosphere.

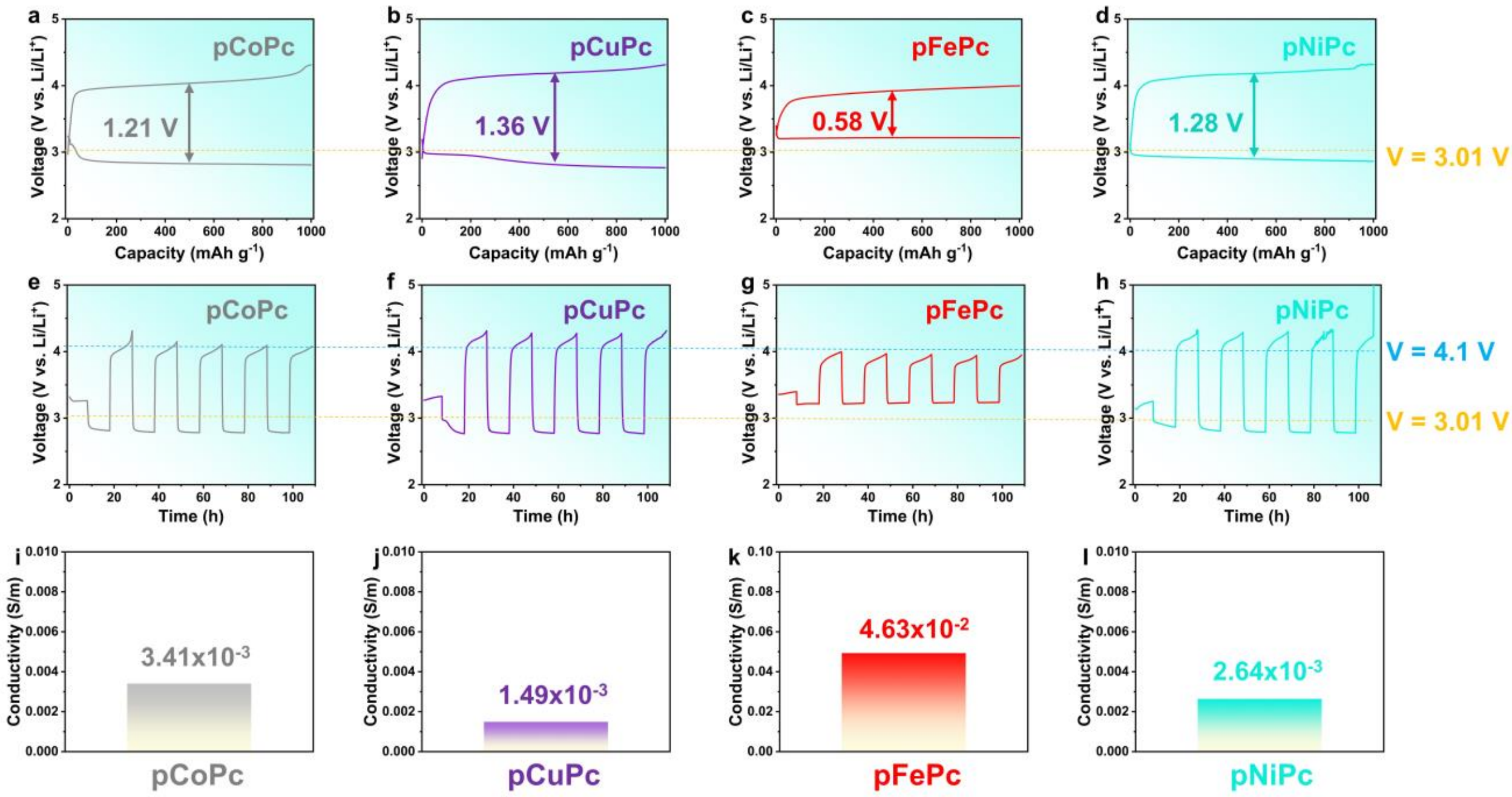

Fig. S7. Electrochemical performance profiles and conductivity measurement results. Comparison of the galvanostatic charge-discharge profiles of Li-CO_2 batteries with (a) pCoPc (b) pCuPc (c) pFePc and (d) pNiPc as solid-state redox mediator within the cut-off capacity of 1000 mAh g^{-1}. Cycling performance of Li-CO_2 batteries with (e) pCoPc (f) pCuPc (g) pFePc and (h) pNiPc as solid-state redox mediator at 100 mA g^{-1}. Electrical conductivity of (i) pCoPc (j) pCuPc (k) pFePc and (l) pNiPc.

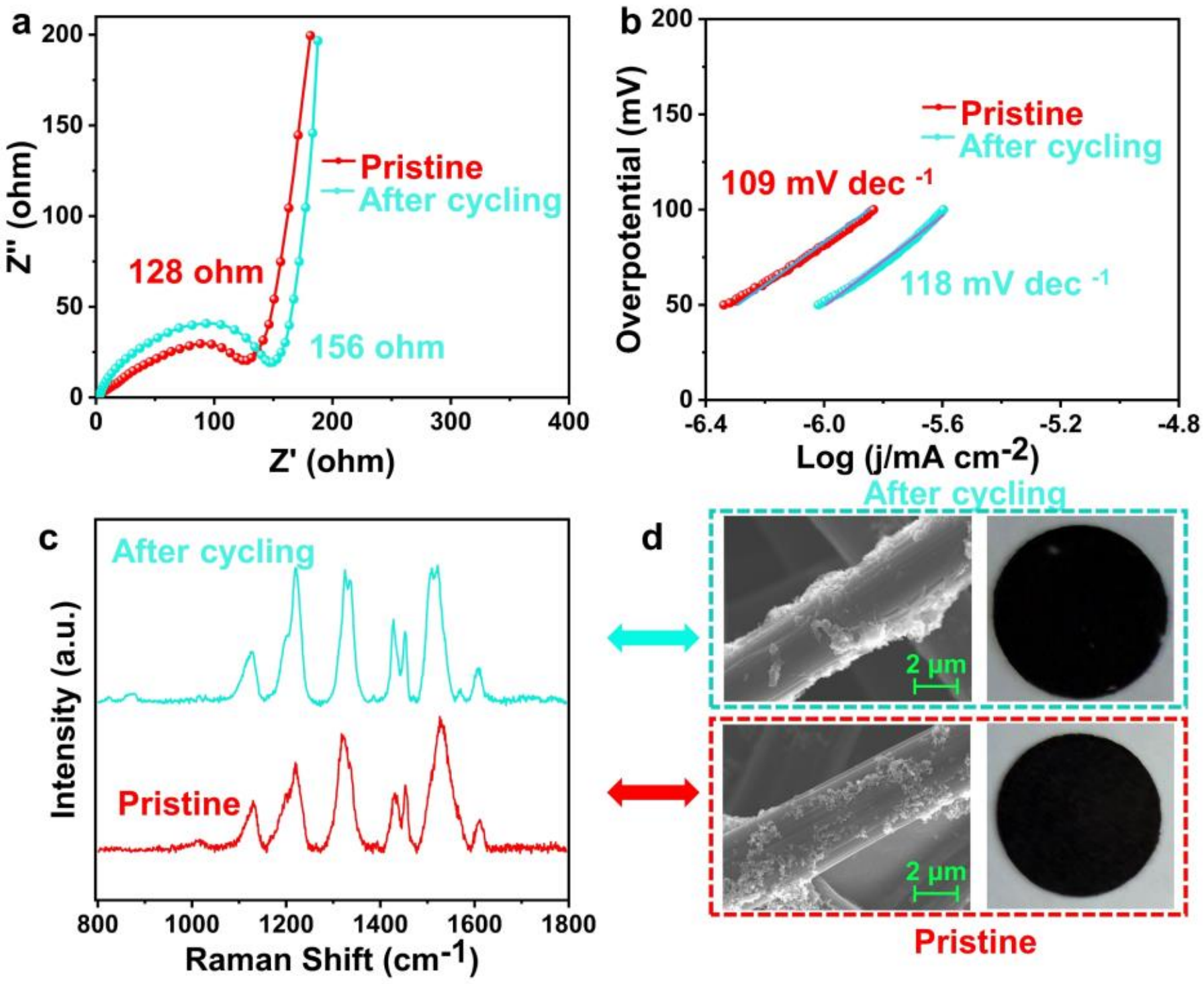

Fig. S8. Characterizations of pFe^{II}Pc electrode. (a) EIS spectra (b) Tafel slope (c) Raman spectroscopy and (d) SEM and optical images of pFe^{II}Pc electrode at different state.

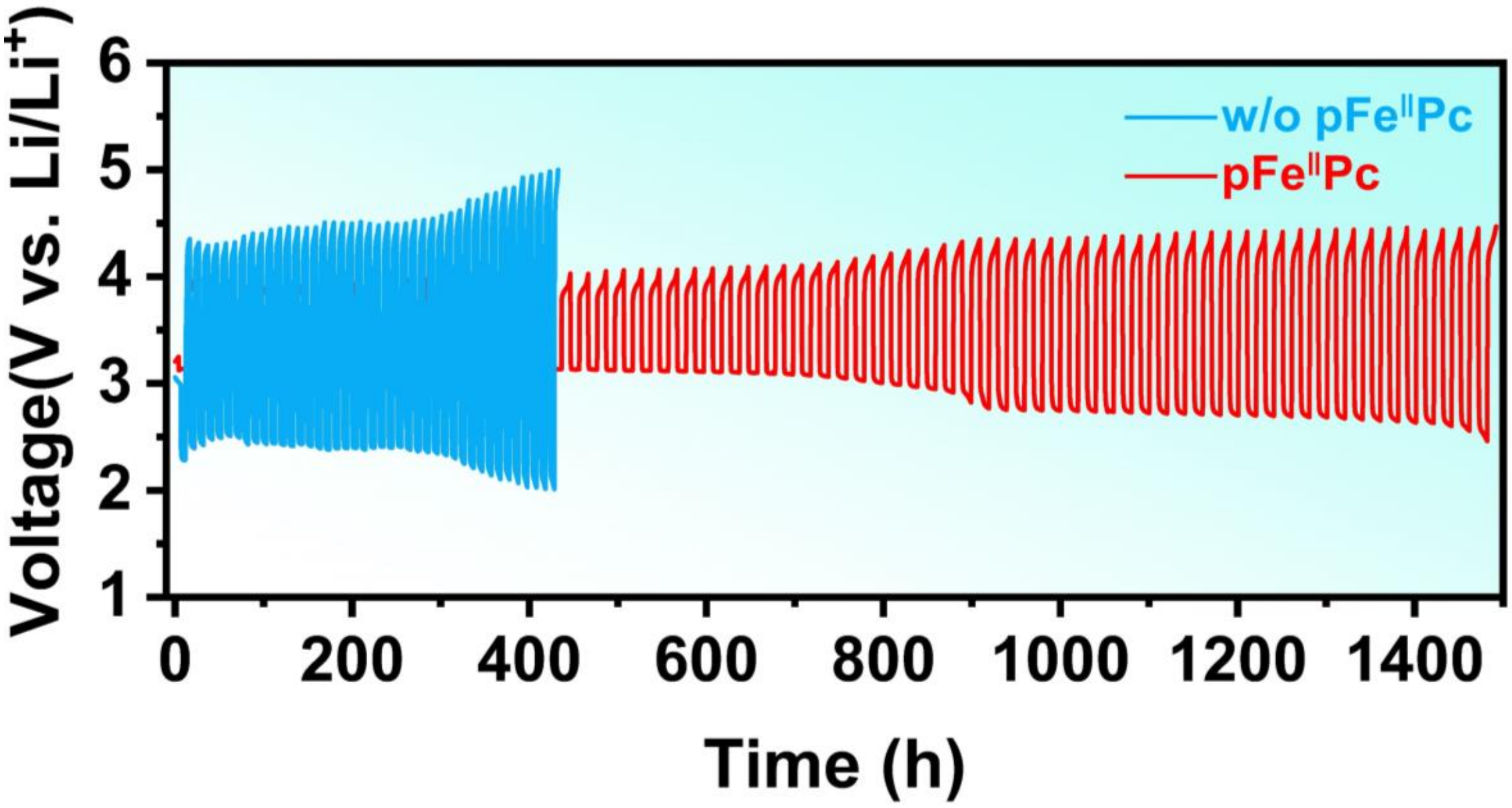

Fig. S9. Voltage-time curves. The cycling performance comparison of Li-CO_2 battery with and without pFe^{II}Pc.

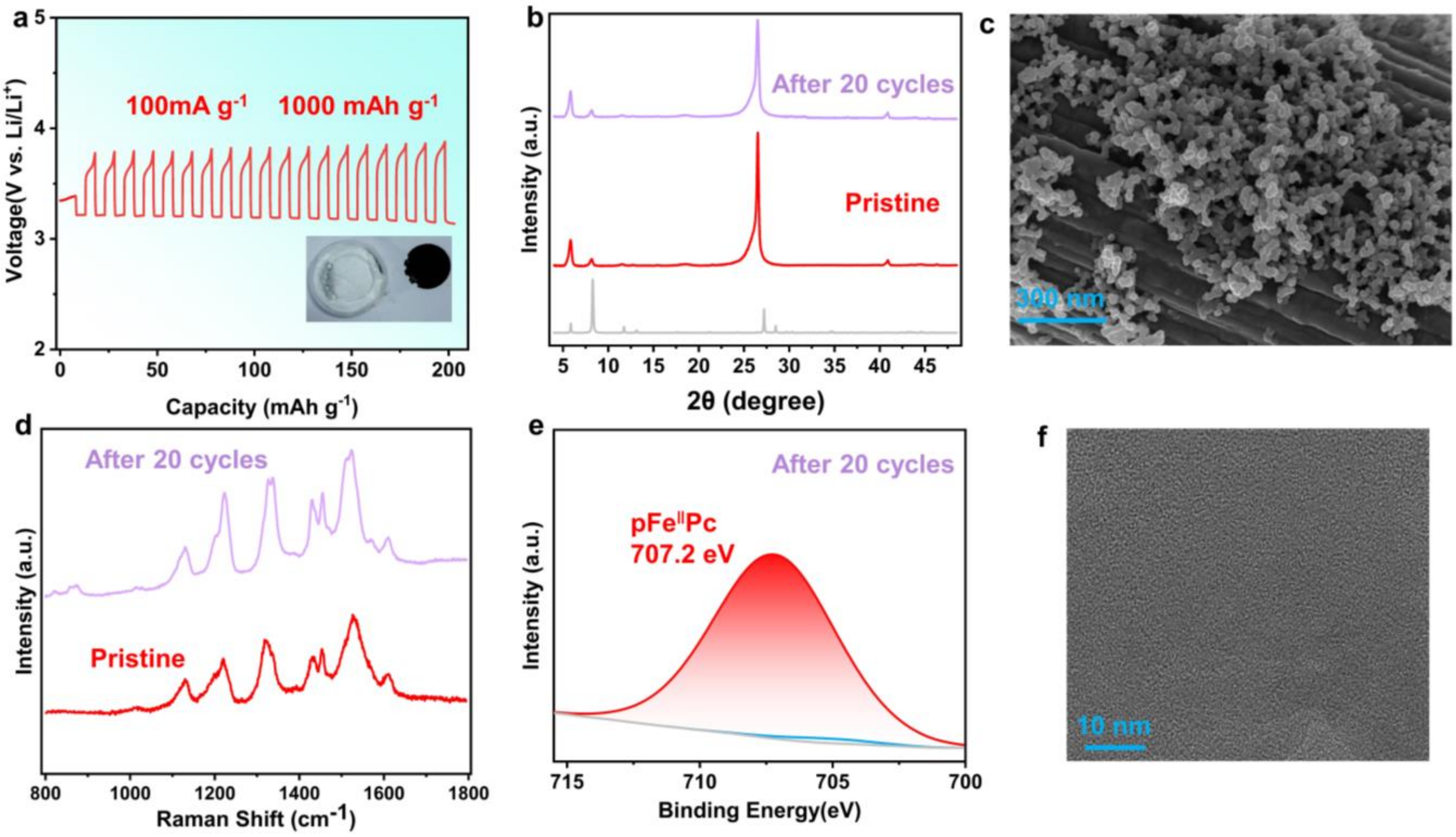

Fig. S10. Cycling curves of Li-CO_2 batteries and characterizations of the pFe^{II}Pc electrode. (a) The charge-discharge curves of the Li-CO_2 battery containing pFe^{II}Pc, cycled for 20 cycles at a current density of 100 mA g^{-1} with a cut-off capacity of 1000 mAh g^{-1}. (b) XRD pattern of the electrode in pristine state and after 20 cycles. (c) SEM image after 20 cycles. (d) Raman spectrum of the electrode in pristine state and after 20 cycles. (e) XPS spectrum of the electrode in pristine state and after 20 cycles. (f) TEM images after 20 cycles.

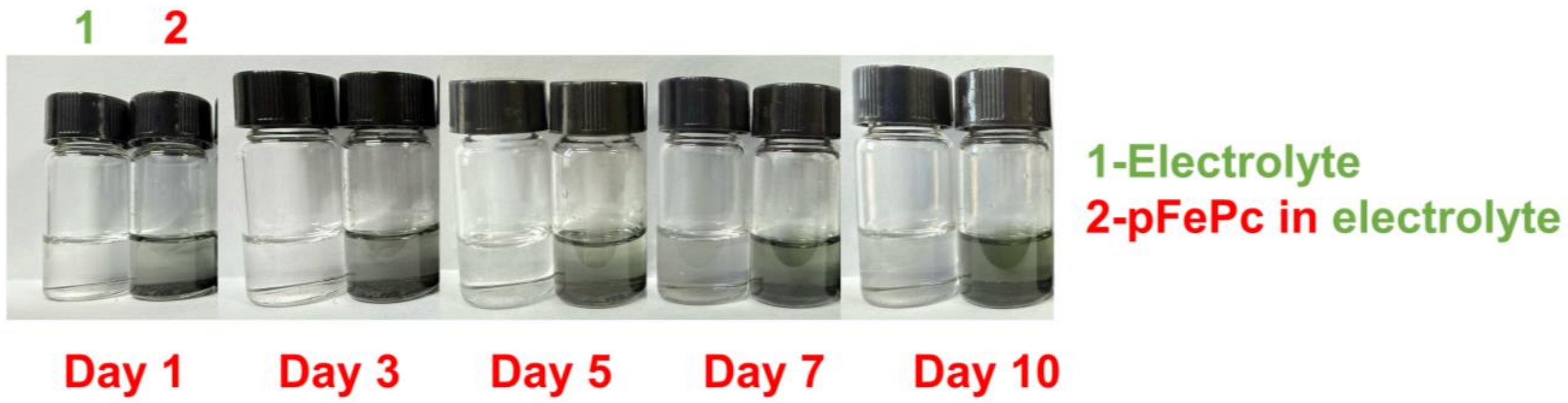

Fig. S11. Optical images. The dissolution behavior of pFe^{II}Pc in LiTFSI/TEGDME solutions at different times.

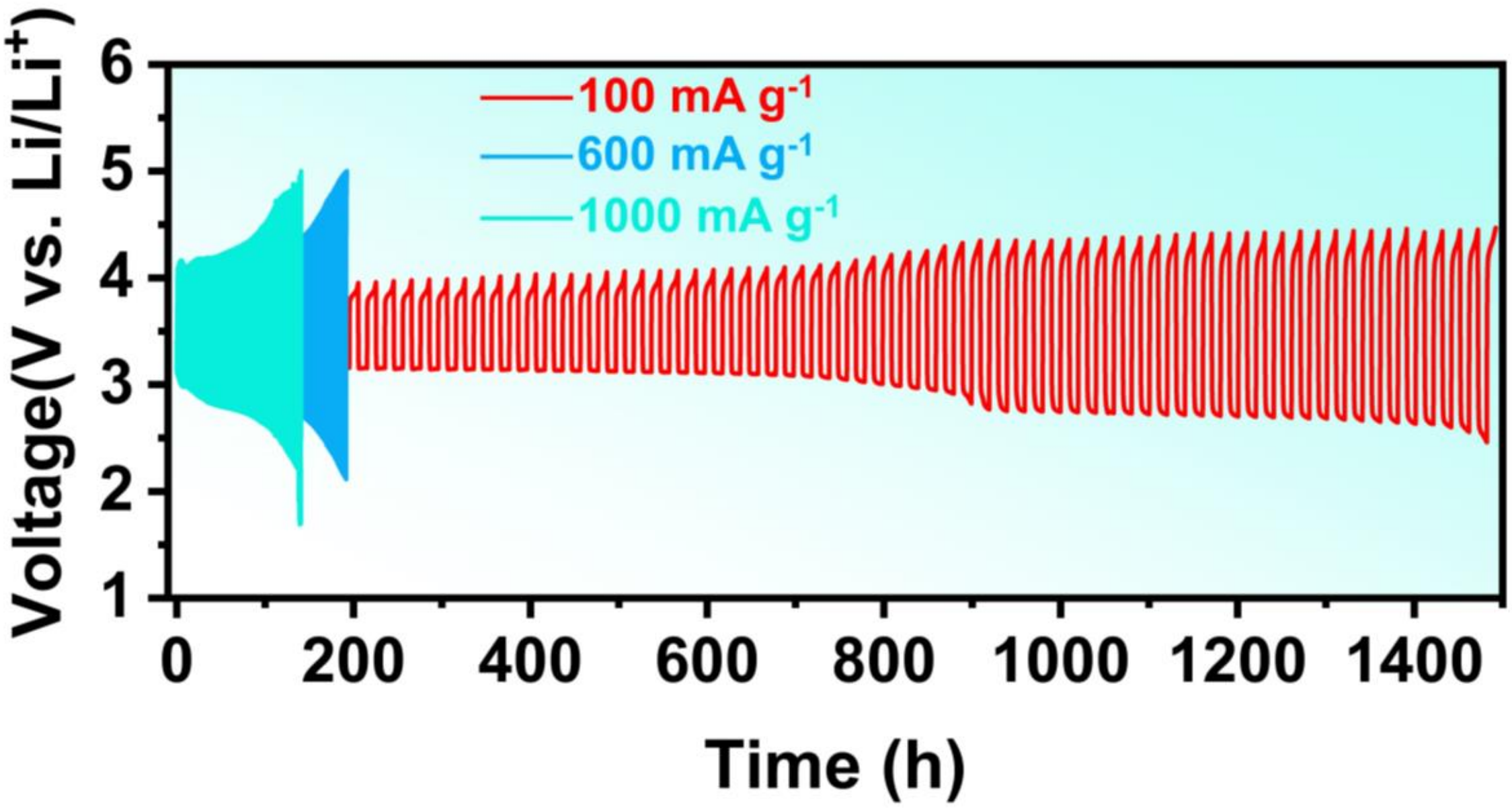

Fig. S12. Cycling curves under various current densities. The cycling performance comparison of Li-CO_2 batteries with pFe^{II}Pc at 100, 600 and 1000 mA g^{-1}, respectively.

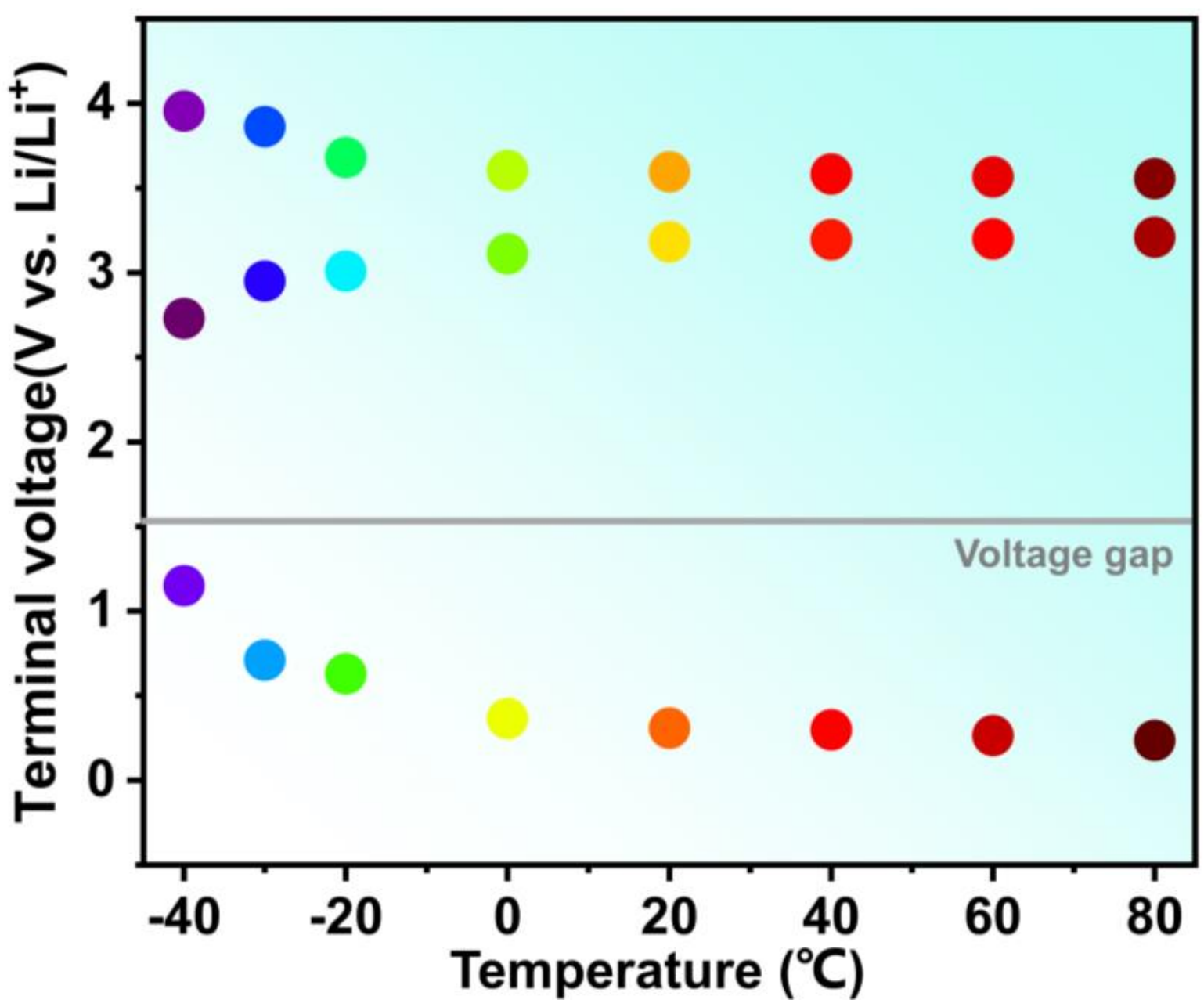

Fig. S13. Voltage gap analysis. Median voltage and voltage gap of Fig. 2H.

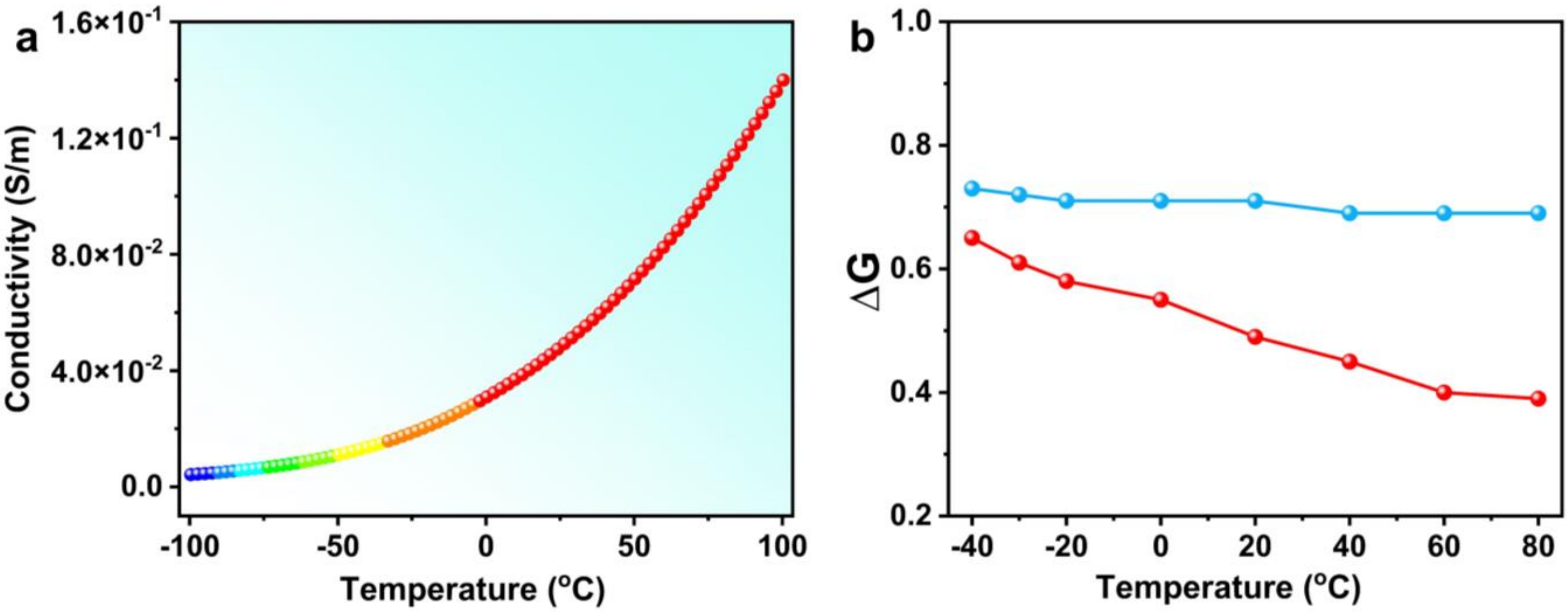

Fig. S14. Results of electrical conductivity and ΔG at different temperatures. (a) The electrical conductivity of pFe^{II}Pc at different temperatures. (b) ΔG of the rate-determining step for the formation of $Li_2C_2O_4$ in the Li-CO_2 battery with pFe^{II}Pc at different temperatures.

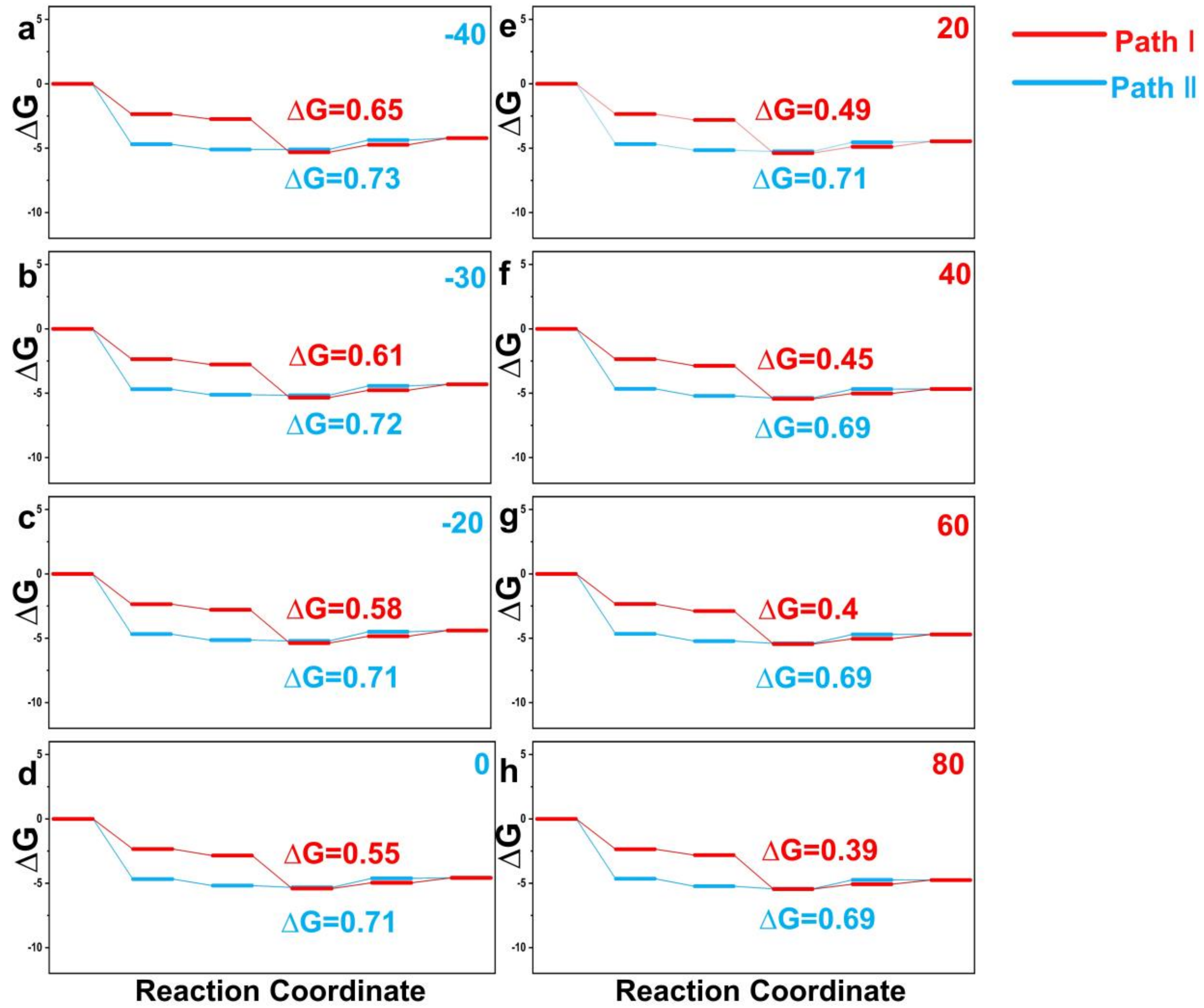

Fig. S15. Free energy of discharge process at different temperatures. Free energy diagrams of different nucleation pathways of $Li_2C_2O_4$ on $C_{32}H_{16}N_8Fe$ and $C_{58}H_{26}N_{16}Fe_2$ at (a) -40 ºC (b) -30 ºC (c)-20 ºC (d) 0 ºC (e)20 ºC (f) 40 ºC (g) 60 ºC (h) 80 ºC.

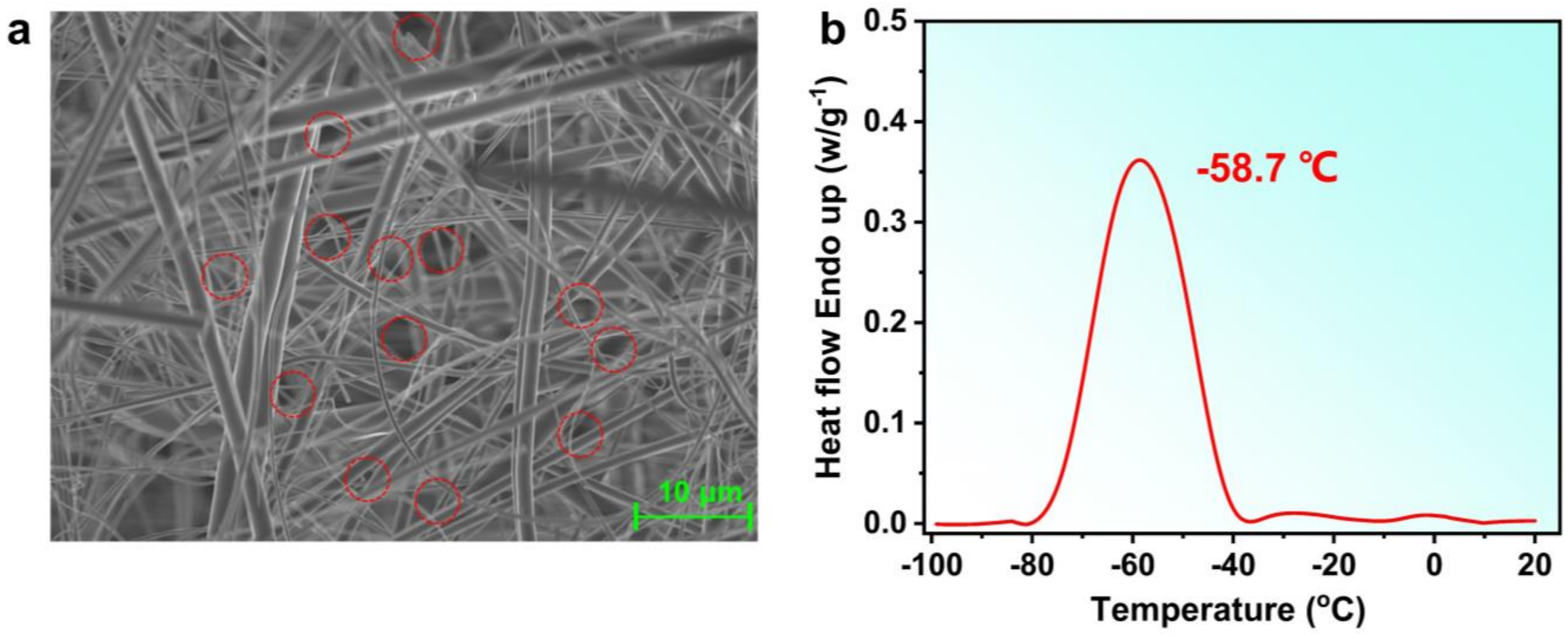

Fig. S16. SEM image and DSC result. (a) SEM image of the separator. (b) The DSC curve of 1.0 M LiTFSI/TEGDME electrolyte in separator.

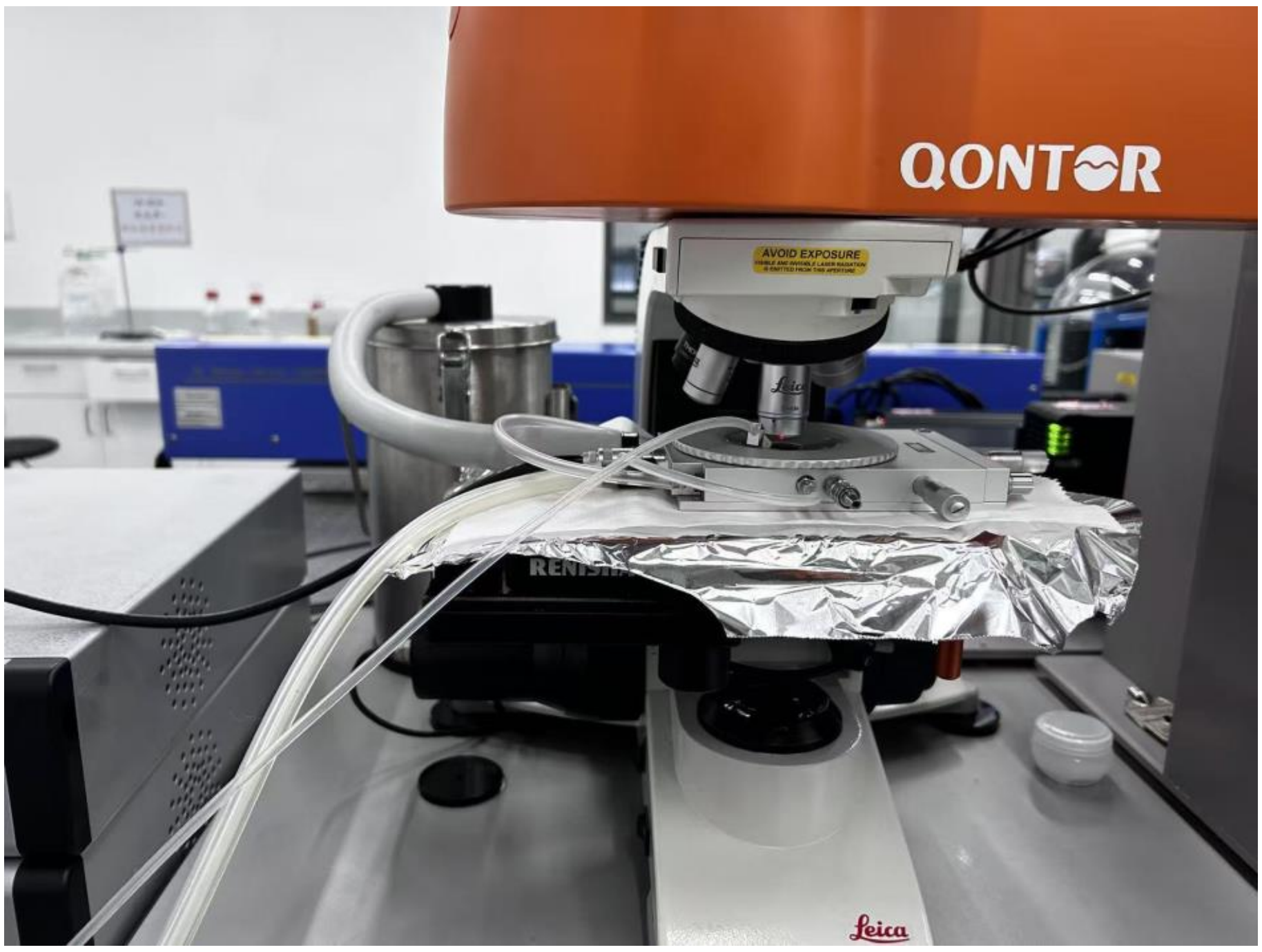

Fig. S17. Raman test setup. Optical image of the variable-temperature in-situ Raman testing process.

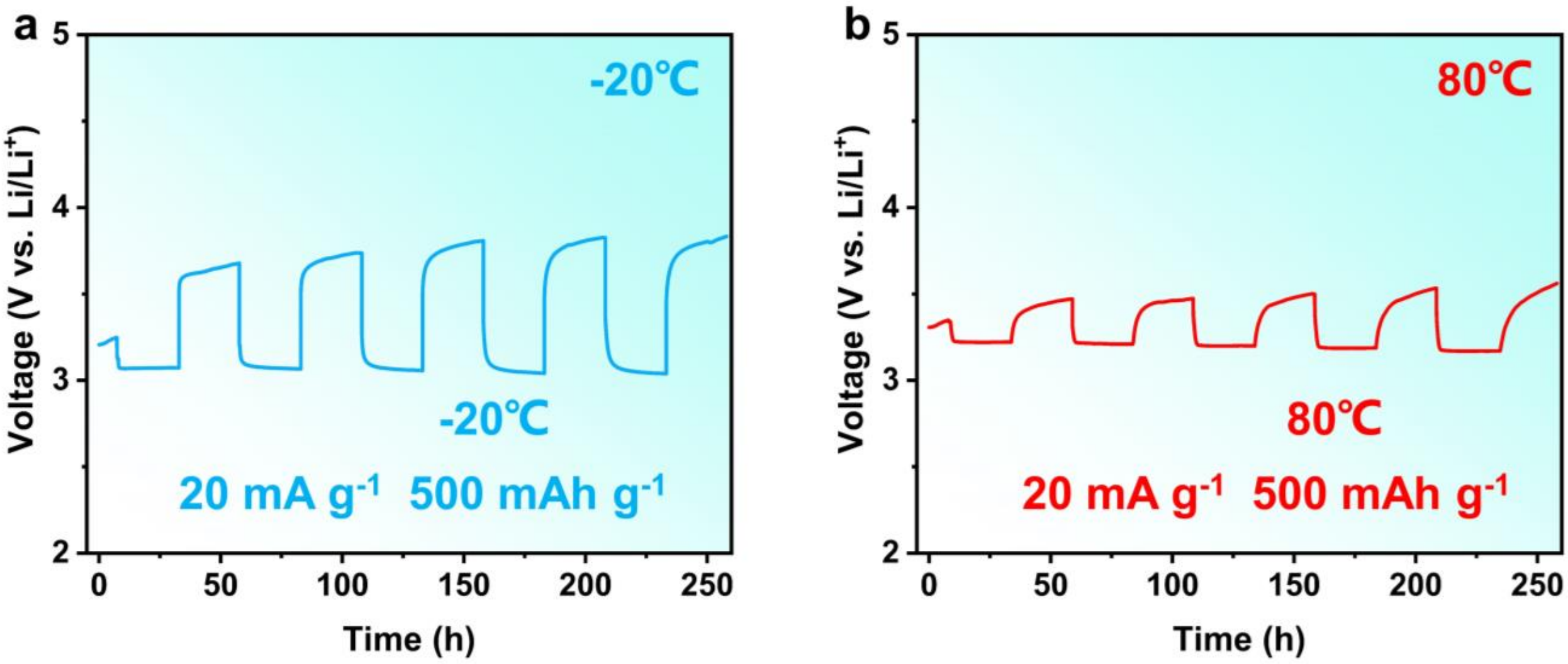

Fig. S18. Cycling curves at different temperatures. The cycling curve of the Li-CO_2 battery with pFe^{II}Pc and a current density of 20 mA g^{-1} at (a) -20 ^oC and (b) 80 ^oC.

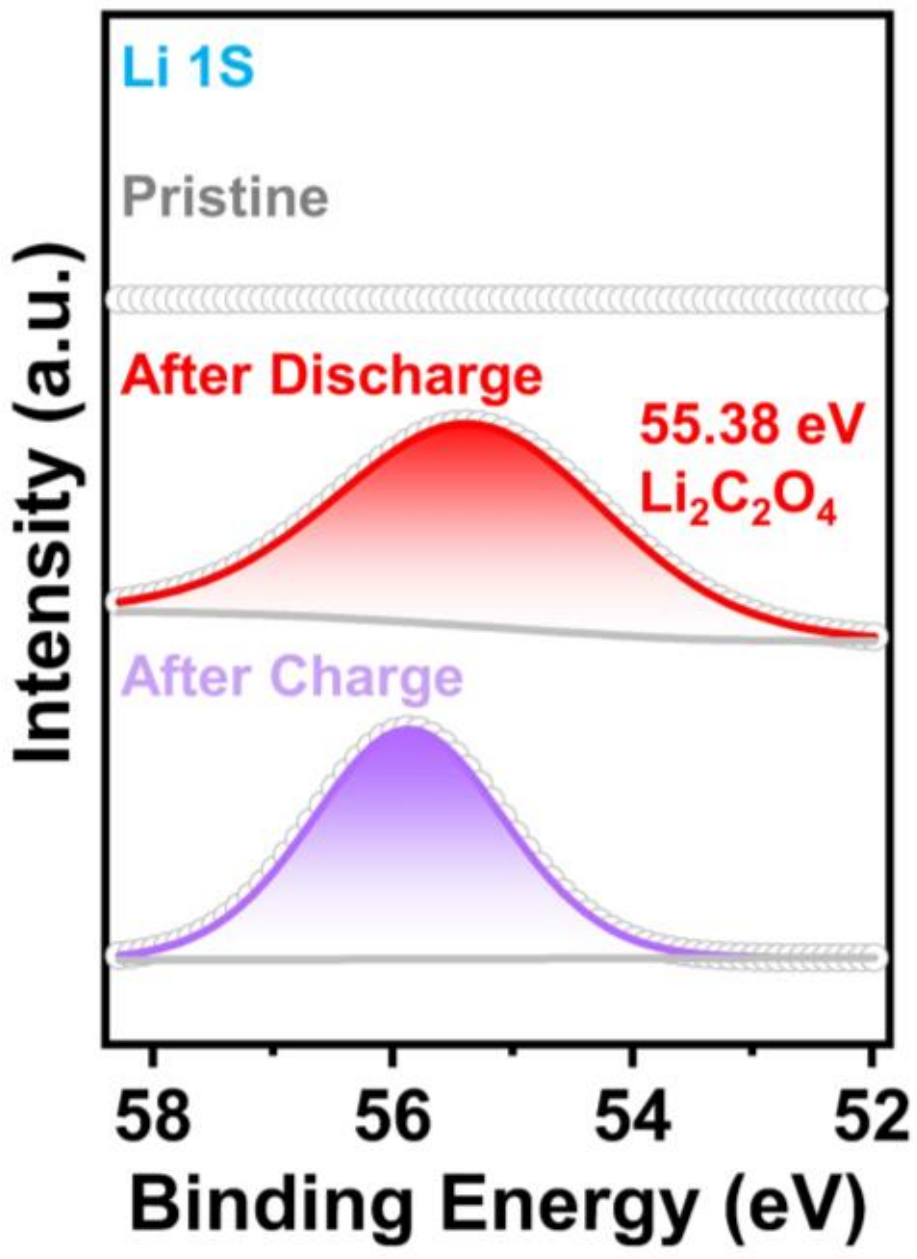

Fig. S19. XPS characterizations. XPS spectra of Li 1s at pristine, after discharge and after charge.

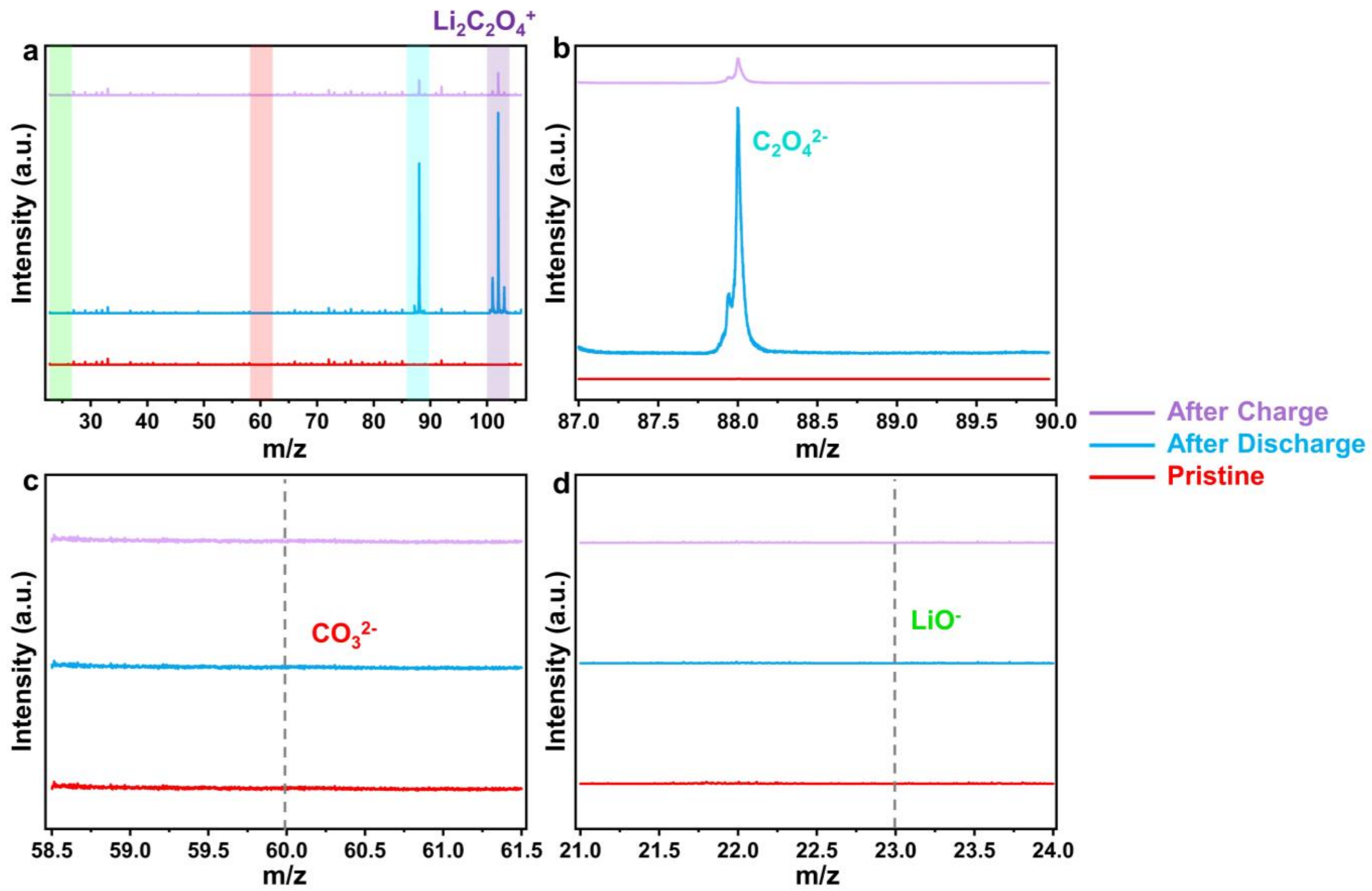

Fig. S20. TOF-SIMS characterizations at different m/z. (a) m/z of 20-110. (b) m/z of 87-90. (c) m/z of 58.5-61.5. (d) m/z of 21-24.

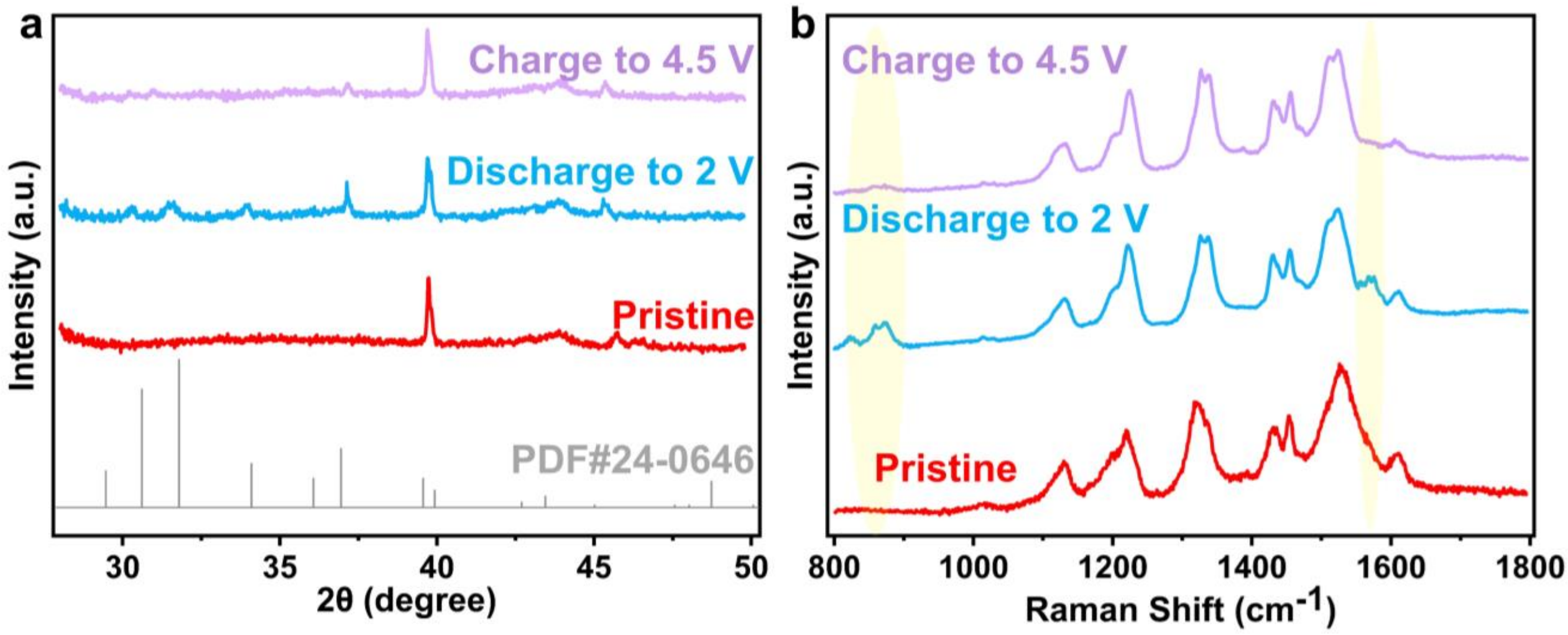

Fig. S21. XRD and Raman characterizations. (a) XRD pattern (b) Raman spectrum of Li-CO_2 battery with pFe^{II}Pc at pristine, after discharge to 2 V and charge to 4.5 V.

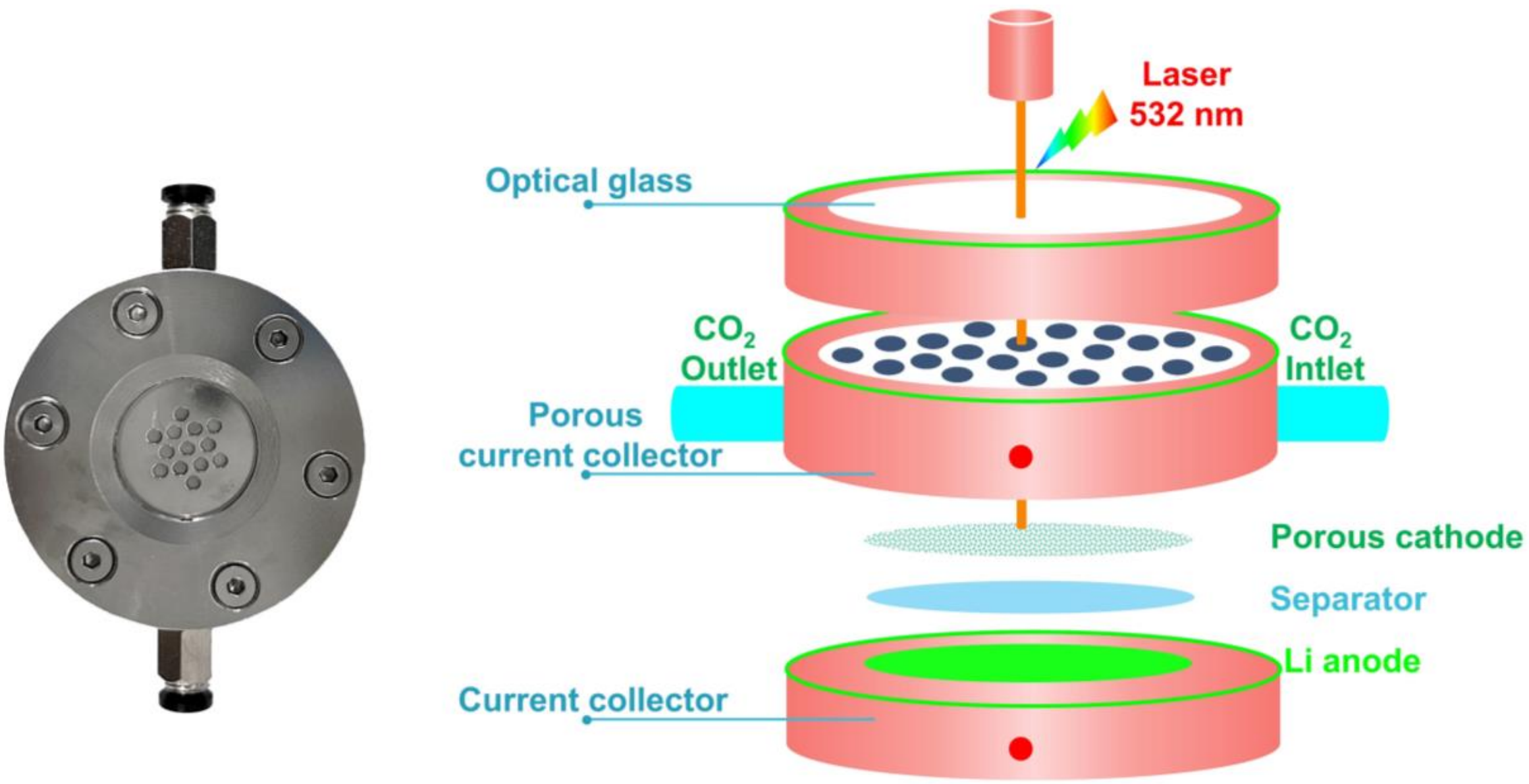

Fig. S22. In-situ Raman testing device. The structure of in-situ Li-CO_2 Raman battery.

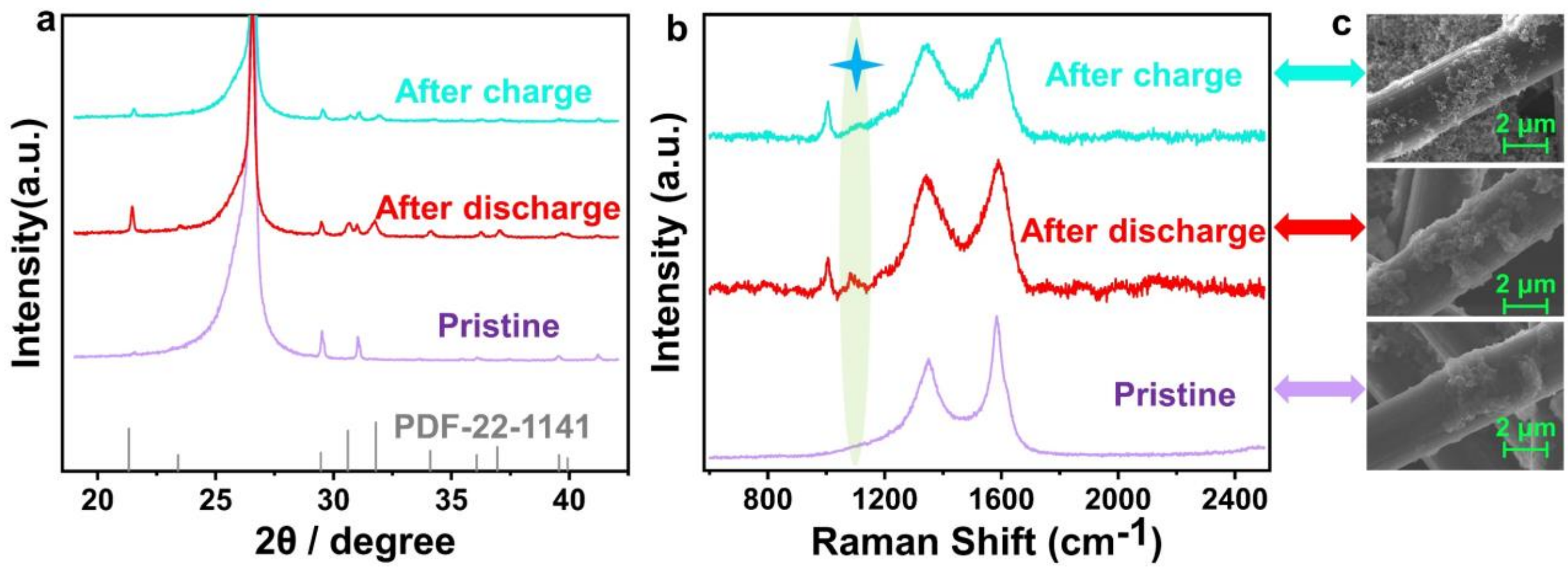

Fig. S23. XRD, Raman characterizations and SEM images. (a) The XRD patterns (b) Raman spectroscopy and (c) SEM images of Li-CO_2 batteries without pFe^{II}Pc at pristine, discharged and recharged, respectively.

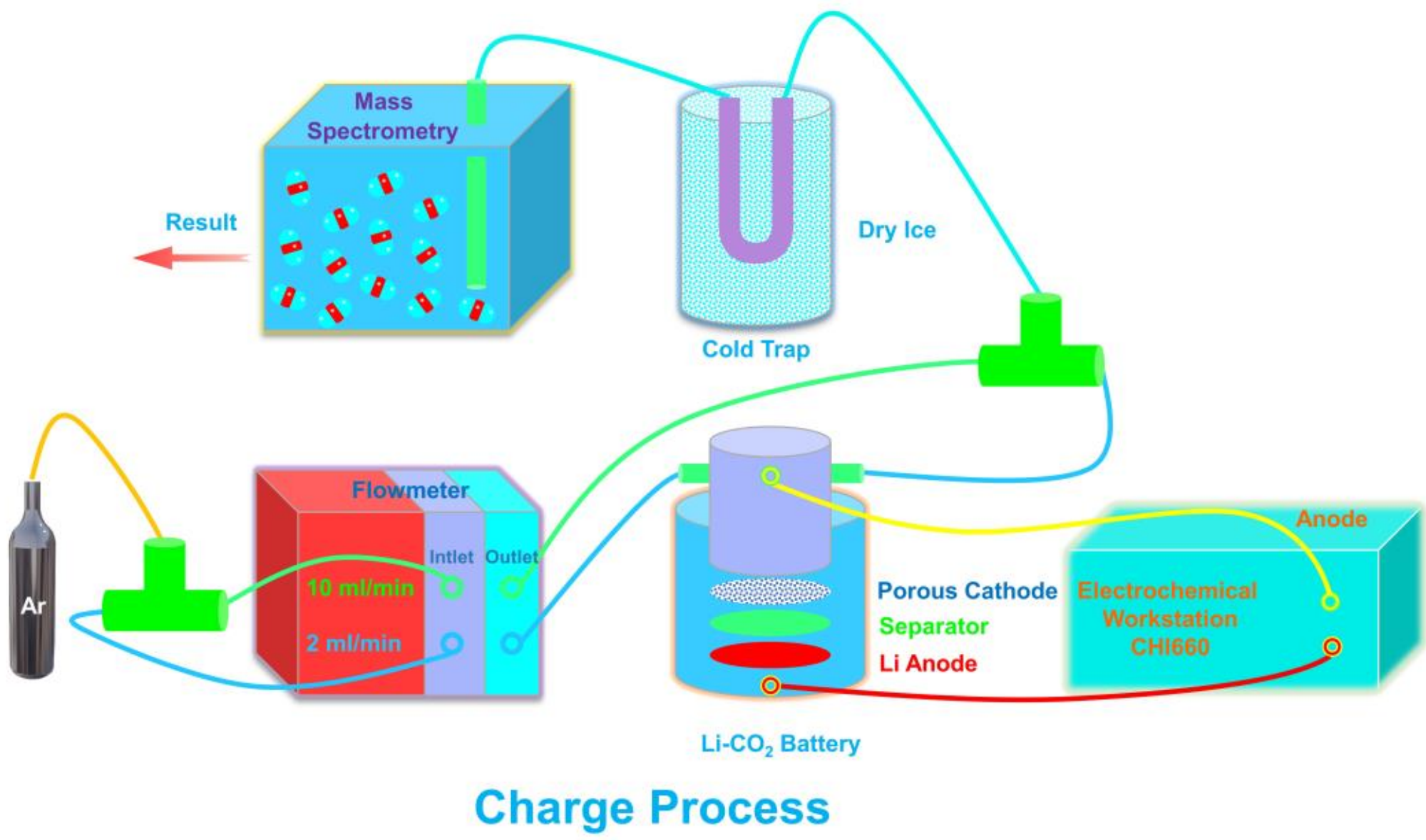

Fig. S24. DEMS measurement schematic. Schematic diagram of the in-situ DEMS for the charge process of the Li-CO_2 battery.

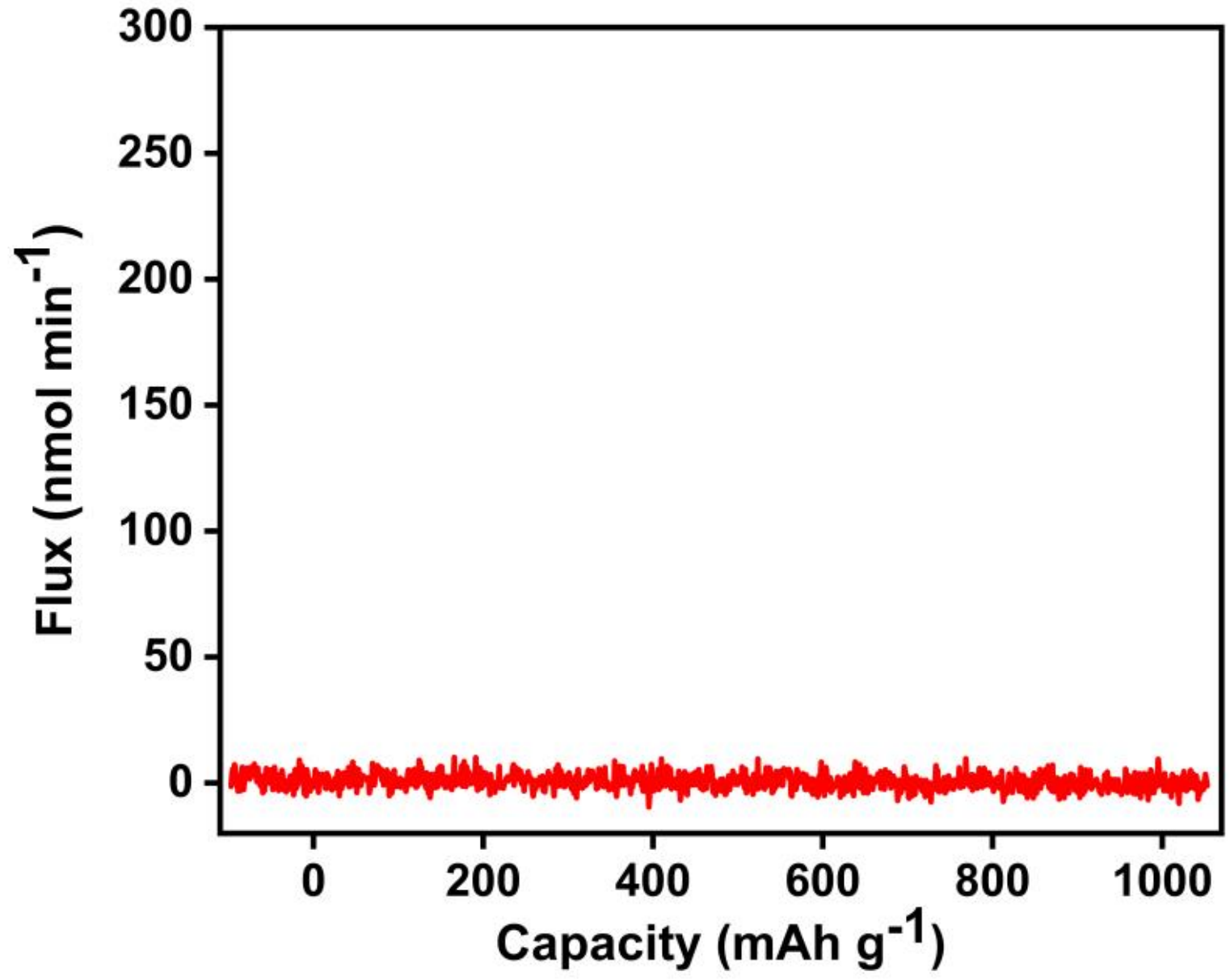

Fig. S25. Result of DEMS measurement. In-situ DEMS profile of Li-CO_2 batteries with pFe^{II}Pc as solid-state redox mediators during charge process of CO.

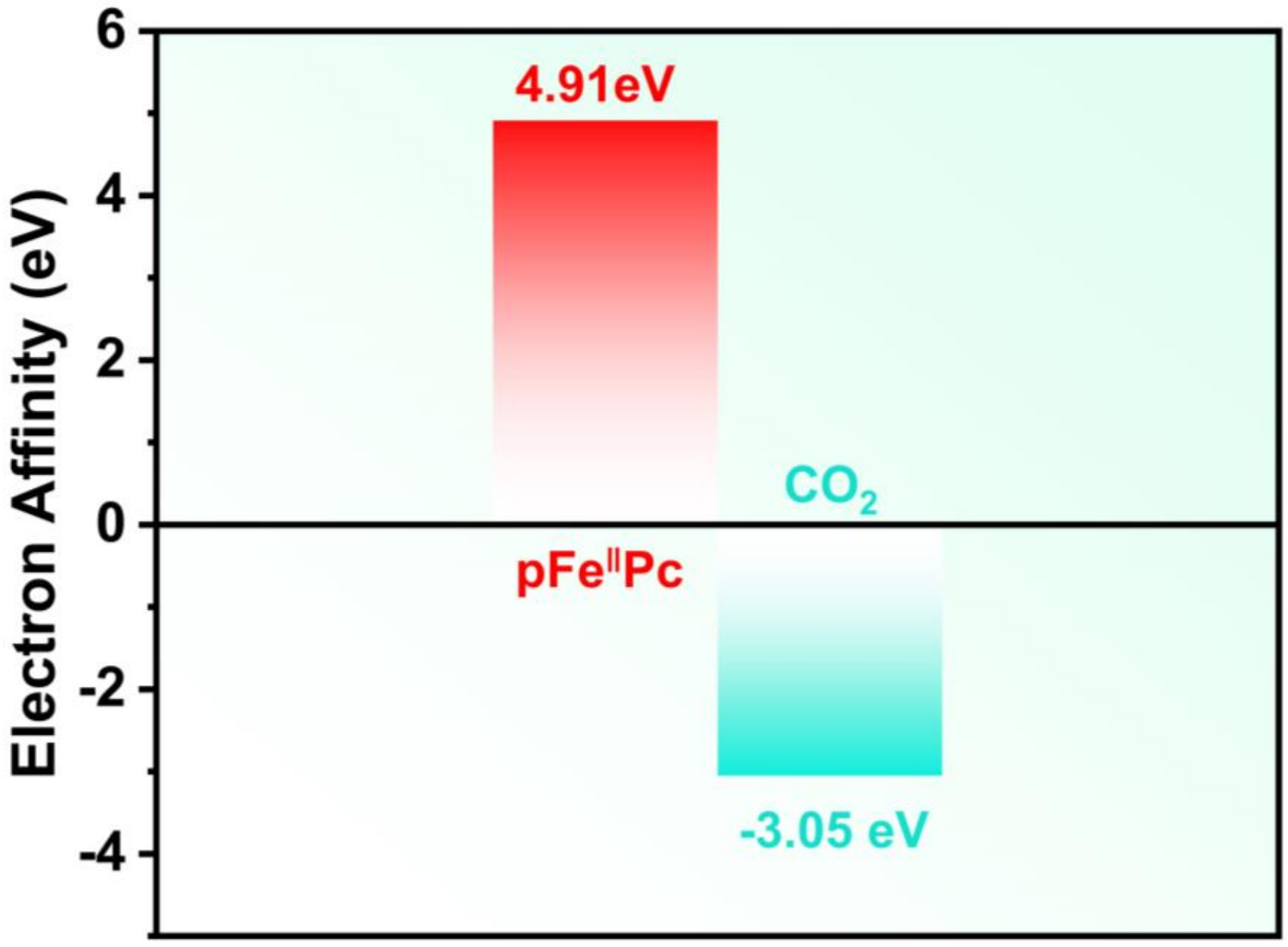

Fig. S26. Calculation results of electron affinities. A comparison of the electron affinities of CO_2 and pFe^{II}Pc.

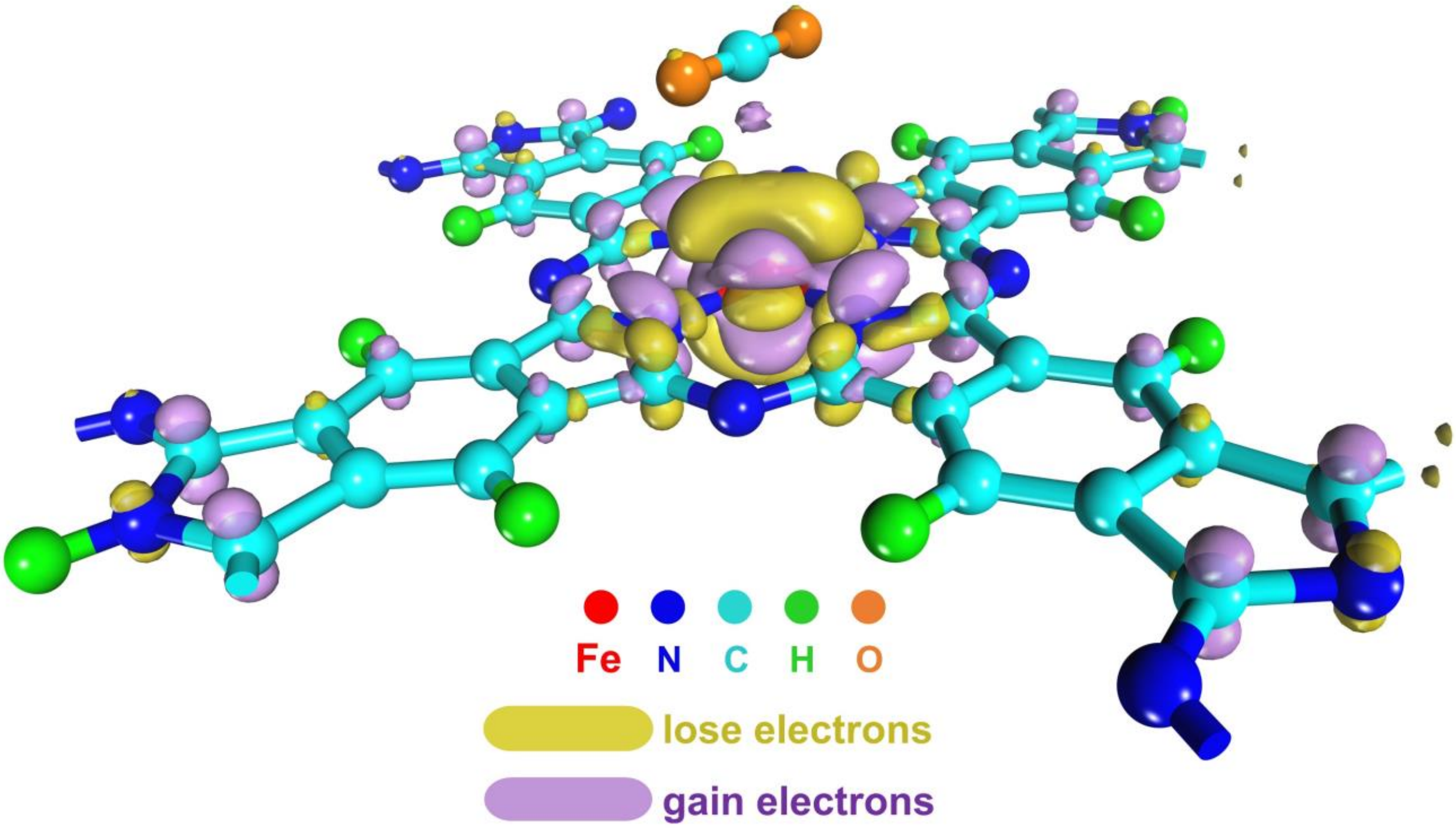

Fig. S27. Electron density difference calculation results. Corresponding electron density difference plot after pFe^{I}Pc adsorb CO_2 (isovalue: 0.005).

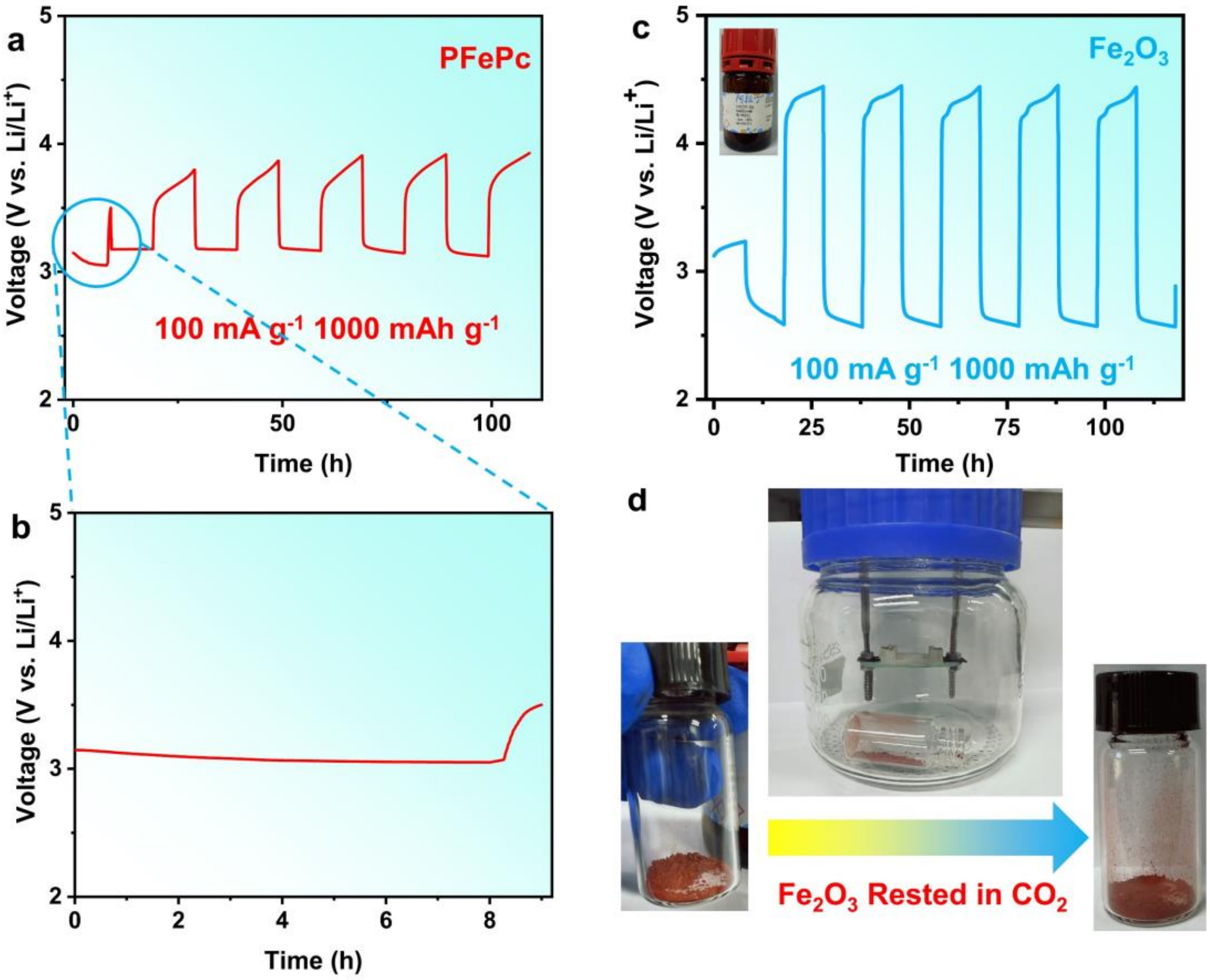

Fig. S28. Electrochemical performance curves and optical images. (a) The charge-discharge curves of the pFe^{II}Pc electrode with the sequence of first charging and then discharging. (b) The enlarged view of Fig. S28a. (c) The charge-discharge curves of the Fe_2O_3 electrode. (d) Fe_2O_3 after being statically placed in CO_2 for 3 days.

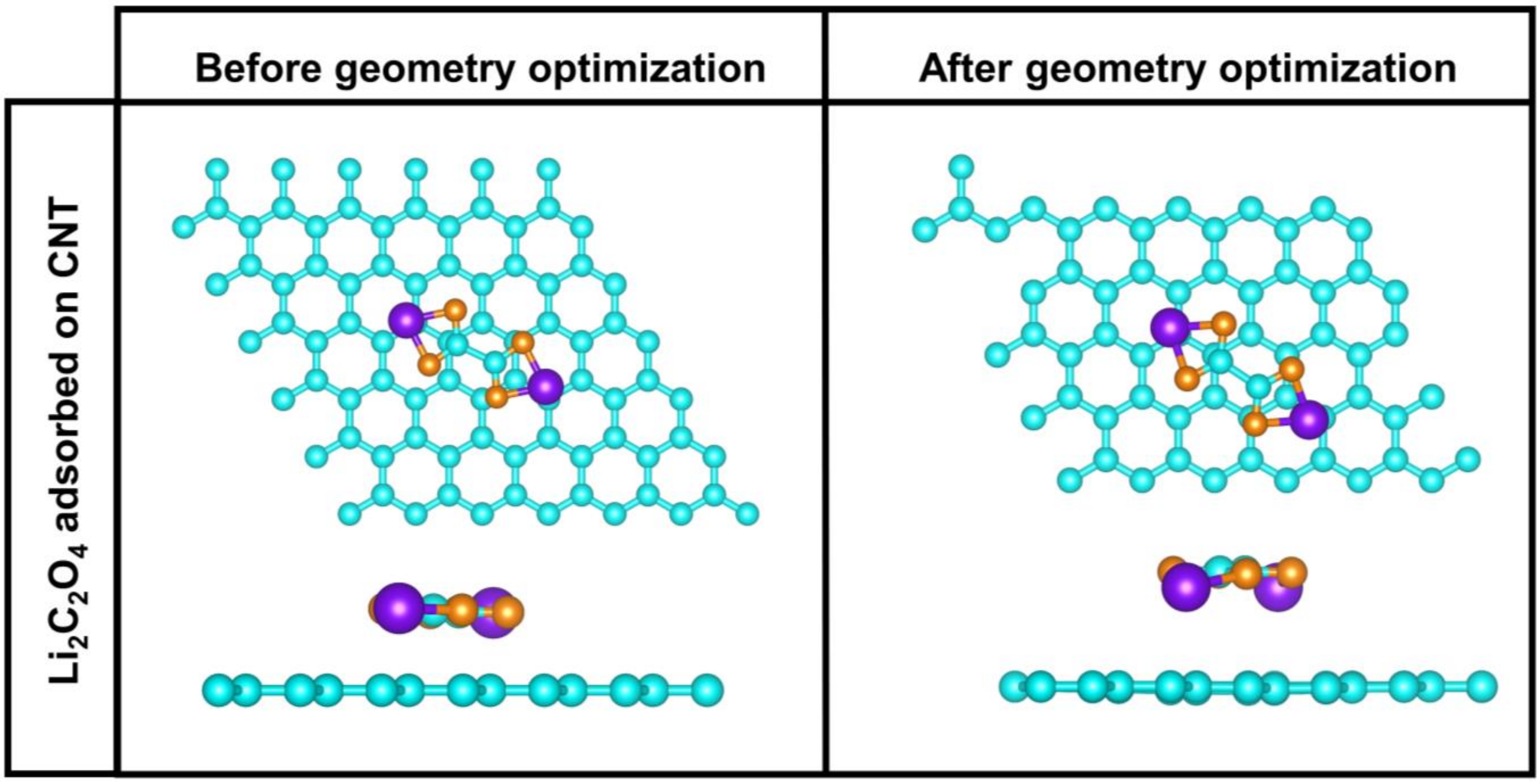

Fig. S29. DFT theoretical calculation model on CNT. Comparison of the $Li_2C_2O_4$ adsorption model on the CNT surface before and after optimization.

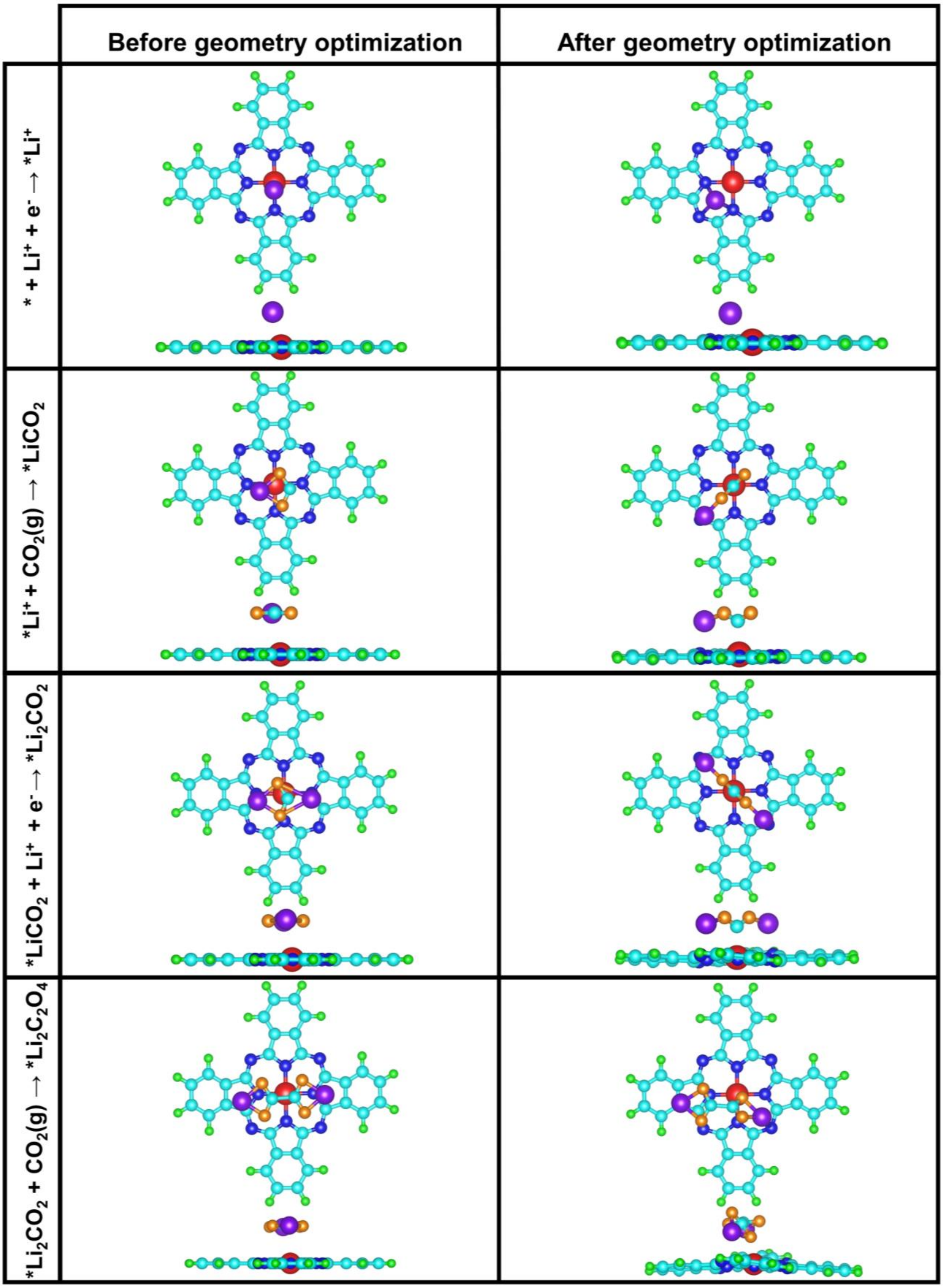

Fig. S30. DFT theoretical calculation model of Path Ⅰ. Comparison of ground state structures before and after optimization for each step in Path Ⅰ.

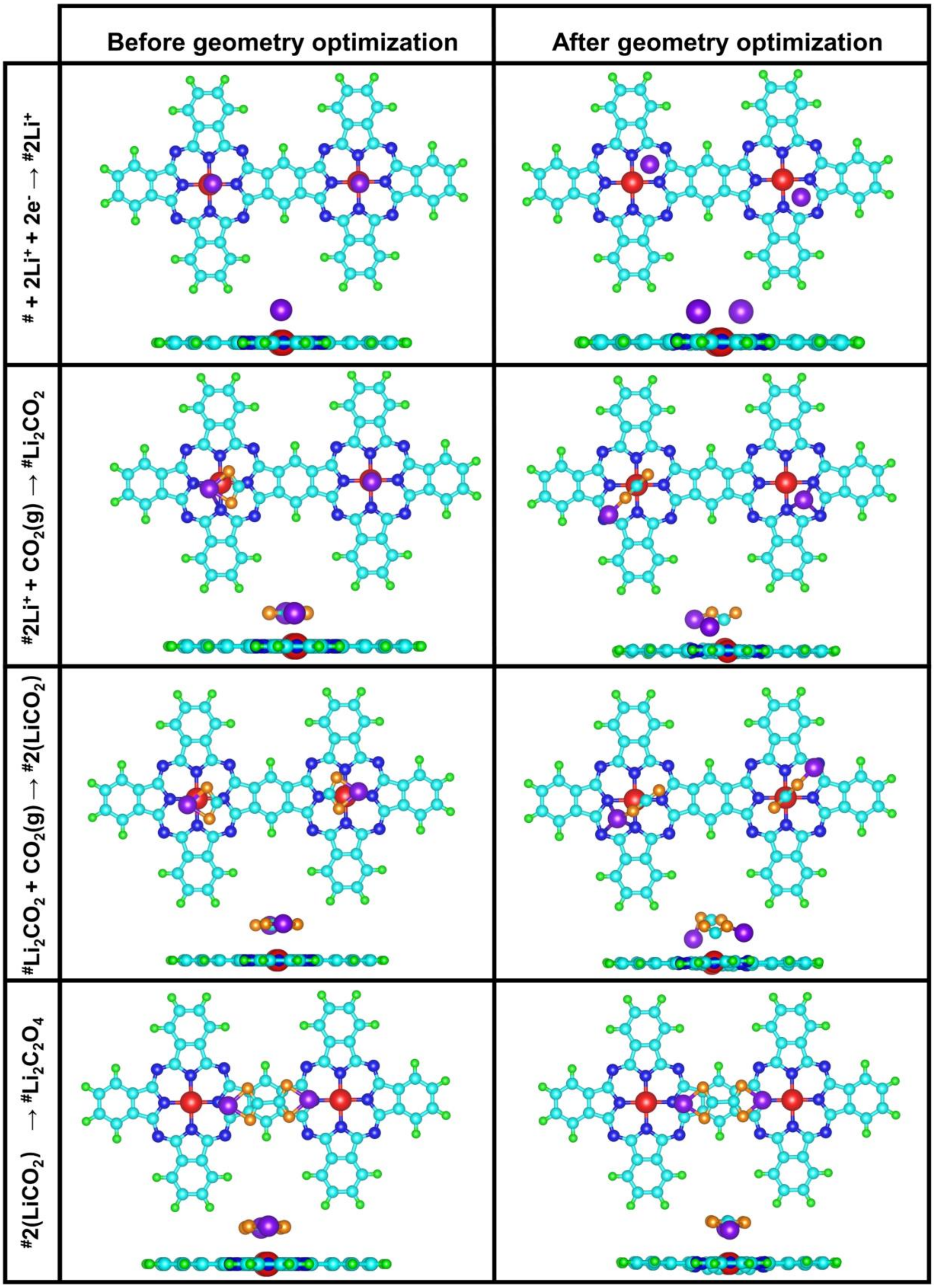

Fig. S31. DFT theoretical calculation model of Path Ⅱ. Comparison of ground state structures before and after optimization for each step in Path Ⅱ.

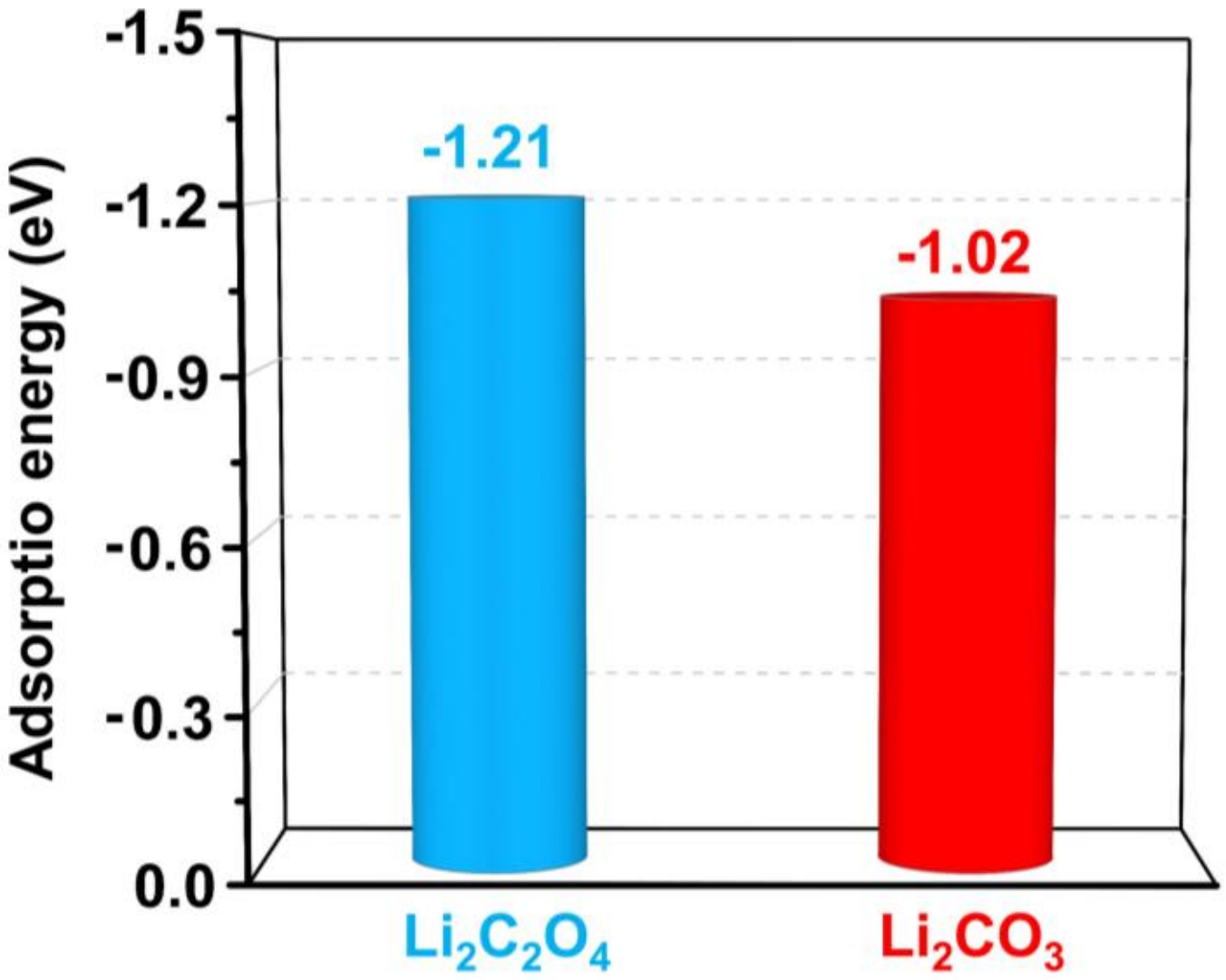

Fig. S32. Adsorption energy comparison. Adsorption energy of $Li_2C_2O_4$ and Li_2CO_3 on $C_{32}H_{16}N_8Fe$.

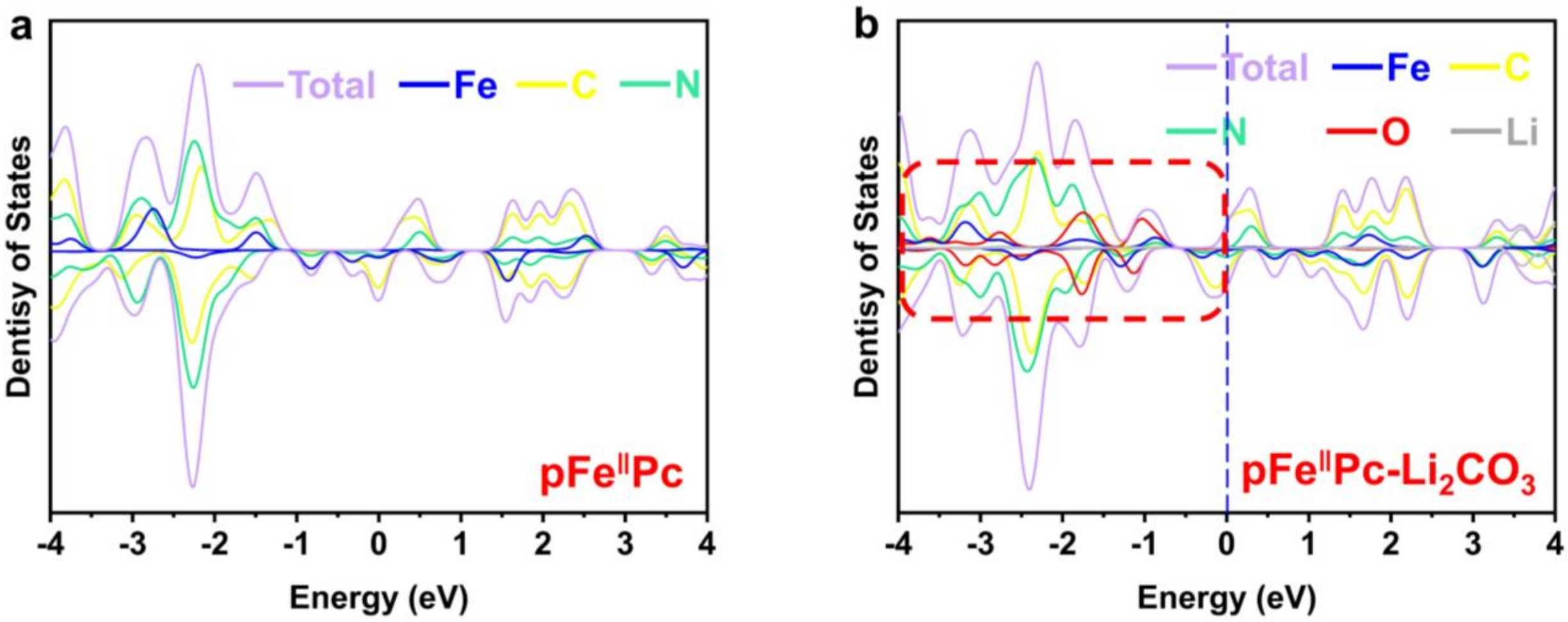

Fig. S33. Theoretical calculation results of PDOS. PDOS of $C_{32}H_{16}N_8Fe$ around the Fermi level (a) $C_{32}H_{16}N_8Fe$ and (b) $C_{32}H_{16}N_8Fe$ after Li_2CO_3 adsorption.
